# Hantaan Virus Infection Induces Both Th1 and ThGranzyme B+ Cell Immune Responses That Associated with Viral Control and Clinical Outcome in Humans

**DOI:** 10.1371/journal.ppat.1004788

**Published:** 2015-04-02

**Authors:** Ying Ma, Bin Yuan, Ran Zhuang, Yusi Zhang, Bei Liu, Chunmei Zhang, Yun Zhang, Haitao Yu, Jing Yi, Angang Yang, Boquan Jin

**Affiliations:** 1 Department of Immunology, the Fourth Military Medical University, Xi’an, China; 2 Institute of Orthopaedics of Xijing Hospital, the Fourth Military Medical University, Xi’an, China; 3 Department of Infectious Diseases of Tangdu Hospital, the Fourth Military Medical University, Xi’an, China; University of Rochester Medical Center, UNITED STATES

## Abstract

Hantaviruses infection causing severe emerging diseases with high mortality rates in humans has become public health concern globally. The potential roles of CD4^+^T cells in viral control have been extensively studied. However, the contribution of CD4^+^T cells to the host response against Hantaan virus (HTNV) infection remains unclear. Here, based on the T-cell epitopes mapped on HTNV glycoprotein, we studied the effects and characteristics of CD4^+^T-cell responses in determining the outcome of hemorrhagic fever with renal syndrome. A total of 79 novel 15-mer T-cell epitopes on the HTNV glycoprotein were identified, among which 20 peptides were dominant target epitopes. Importantly, we showed the presence of both effective Th1 responses with polyfunctional cytokine secretion and ThGranzyme B^+^ cell responses with cytotoxic mediators production against HTNV infection. The HTNV glycoprotein-specific CD4^+^T-cell responses inversely correlated with the plasma HTNV RNA load in patients. Individuals with milder disease outcomes showed broader epitopes targeted and stronger CD4^+^T-cell responses against HTNV glycoproteins compared with more severe patients. The CD4^+^T cells characterized by broader antigenic repertoire, stronger polyfunctional responses, better expansion capacity and highly differentiated effector memory phenotype(CD27^-^CD28^-^CCR7^-^CD45RA-CD127^hi^) would elicit greater defense against HTNV infection and lead to much milder outcome of the disease. The host defense mediated by CD4^+^T cells may through the inducing antiviral condition of the host cells and cytotoxic effect of ThGranzyme B^+^ cells. Thus, these findings highlight the efforts of CD4^+^T-cell immunity to HTNV control and provide crucial information to better understand the immune defense against HTNV infection.

## Author Summary

Hantaan virus (HTNV), the prototype of Hantavirus genus, is a rodent-borne pathogen that causes human hemorrhagic fever with renal syndrome, with mortality rate of approximately 15% in Asia. The efforts of our immune system to defend against HTNV are important for clearance of the infection. However, the interaction between CD4^+^T-cell immunity and HTNV infection in humans is not known. Based on the novel T-cell epitopes we defined on HTNV glycoprotein in Chinese Han population, we confirmed that HTNV glycoprotein could induce vigorous and extensive CD4^+^T-cell response in humans. For the first time, we showed that both Th1 and ThGranzyme B^+^ cell responses involved in defense against HTNV infection and inversely correlated with plasma viral load and disease outcome. Additionally, we found that CD4^+^T cells characterized by broader antigenic repertoire, polyfunctional cytokine secretion, stronger expansion and highly differentiated effector memory phenotype always lead to much milder outcome of the disease, maybe through inducing antiviral condition of host cells and cytotoxic effect of ThGranzyme B^+^ cells. Our results add weight to the contribution of CD4^+^T cells in disease control after HTNV infection in humans, which may greatly advance the understanding about how HTNV interact with their host organisms.

## Introduction

During the past decade, hantaviruses, belonging to the *Bunyaviridae* family, have gained worldwide attention as widespread emerging zoonotic pathogens [1–2]. Two clinical conditions of human hantavirus infections have been recognized worldwide: 1) hemorrhagic fever with renal syndrome (HFRS) primarily reflecting infections with Hantaan virus (HTNV) in Asia, Dobrava and Puumala (PUUV) viruses in Europe, and Seoul virus worldwide [3–4] and 2) hantavirus pulmonary syndrome (HPS) primarily reflecting infections with Sin Nombre (SNV) and Andes (ANDV) viruses in North and South America, respectively [5]. Globally hantaviruses might cause as many as 200,000 cases of human disease per year, of which more than a half of the disease cases are fulminant HFRS [6]. A total of 100,868 cases were reported during 2005–2012 in mainland China, where HFRS cases, primarily reflecting infections with the prototype member HTNV strain, account for 90% of the total global cases, with a case-fatality rate as high as 15% [7–9]. Moreover, the recent outbreak of HPS in Yosemite National Park in California, USA, showed a higher case-fatality rate of approximately 37%, thereby raising the concerns of the World Health Organization [10]. Because of the high morbidity and mortality, poorly understood disease pathogenesis, and potential use of pathogenic hantaviruses as weapons for bioterrorism, the Biological Weapons Convention has classified these viruses as Category pathogens; therefore, better understanding the immune mechanism against HTNV infection is of priority for global public health and safety.

The antigenicity of hantaviruses largely depends upon two structure proteins, nucleocapsid protein (NP) and envelope glycoprotein. HTNV-NP has been demonstrated to be highly immunogenic and conservative, inducing vigorous cellular and humoral immune responses in humans [11]. HTNV glycoprotein, heterodimers of mature glycoprotein Gn and Gc, is not only responsible for receptor binding and membrane fusion, but also considered as the main source of neutralizing antibody production [12–13]. T-cell immunity is a critical factor for protection from virus infections in humans. The T-cell epitopes on NP of hantaviruses and associated immune responses have been well-characterized [14–17]. We have previously identified eight novel HTNV-NP cytotoxic T cell (CTL) epitopes with different HLA restrictions [18–19] and showed that HTNV-NP-specific T-cell responses might reduce the risk of progression to acute renal failure [18,20]. Indeed, hantavirus Gn/Gc-specific T-cell responses have also been observed. Several CD8^+^T-cell clones recognizing epitopes on Gn/Gc have been obtained from the blood of HFRS patients infected with PUUV, ANDV or SNV [14,21–23]. Gn specific long-lived effector memory T-cell responses might contribute to protective immunity in ANDV-infected patients [22]. Similarly, protective immunity elicited through infection with recombinant HTNV-Gn/Gc in murine models have also been demonstrated [24]. Therefore, hantavirus Gn/Gc could also serve as a potent immunogen that induces T-cell responses. However, the T-cell epitopes on HTNV-Gn/Gc have not been identified, and the specific responses to these immunogens remain largely unknown.

Recent studies have demonstrated that CD4^+^T cells are also essential for the effective clearance of viral infections [25–26]. Virus-specific CD4^+^T cells are important for initiating and maintaining immunity against most viruses through a variety of mechanisms. (1) The rapid production and secretion of cytokines from CD4^+^T cells during a viral infection, which could induce an antiviral state in the host, indirectly priming the CD8^+^T-cell response [27] and facilitating antigen-specific antibody production [28]. (2) Subsequently, CD4^+^T cells are required to maintain and modulate effective CTL responses and neutralizing antibody [29] and develop long-term memory B and CD8^+^T cells. (3) CD4^+^T cells can also mediate direct cytotoxicity [29–30] and recruit innate or antigen-specific effector cells to the site of viral replication [31]. Enhancement of antiviral immunity through cytotoxic CD4^+^T cells has been proved [32]. The cytotoxic CD4^+^CTLs may represent a distinct subset of effector cells defined by the absence of the master regulator ThPOK [33]. Despite the importance of CD4^+^T cells during virus infection, CD4^+^T-cell immunity towards hantaviruses remains limited. To date, little information regarding the CD4^+^T-cell epitopes on the Gn/Gc of hantaviruses has been reported. Moreover, the role of CD4^+^T cells in hantavirus clearance in humans remains unclear.

To investigate human CD4^+^T-cell immunity to HTNV, the T-cell epitopes on HTNV-Gn/Gc and the specific CD4^+^T-cell responses were evaluated in a large cohort of HFRS patients from Chinese Han population. Herein, we found that HTNV-Gn/Gc could induce vigorous CD4^+^T-cell responses characterized by a broad antigenic repertoire, enhanced expansion, polyfunctional cytokine secretion and functional phenotypes with both T helper 1 (Th1) and ThGranzyme B^+^ (ThGzmB^+^) cell effects. Importantly, HTNV-Gn/Gc-specific CD4^+^T-cell responses were obviously associated with viral control and clinical outcome. Thus, this study is the first to reveal the properties of a primary, protective CD4^+^T-cell immune response to HTNV infection in humans, providing a foundation for understanding host immune responses to HTNV infections.

## Results

### Distribution of T-cell epitopes and the pattern of T-cell reactivity across the HTNV glycoprotein

**Fig 1:**
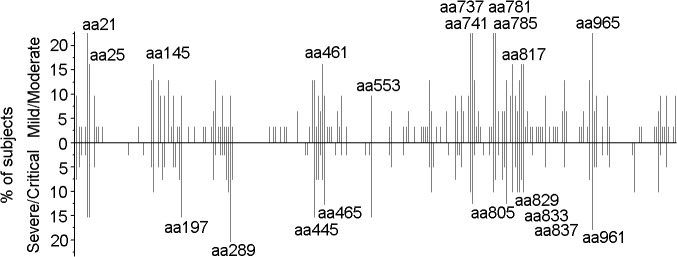
Distribution of 15-mer T-cell epitopes and frequency of recognition over the HTNV-Gn/Gc full sequence. Each *bar* represents a distinctive HTNV-Gn/Gc T-cell epitope, with the length of the *bar* indicating the percentage of subjects targeting this epitope. *Bars* above the line correspond to responses at acute stage in the cohort with mild or moderate infection (n = 31), and the *bars* below the line represent responses detected in acute phase in persons with severe or critical infections (n = 39). The 20 epitopes with the start amino acid number of each peptide indicated were defined as the immunodominant epitopes, which were frequently recognized (≥10% of the total positive responding subjects, n = 70) in the patients. aa, amino acid.

**Fig 2:**
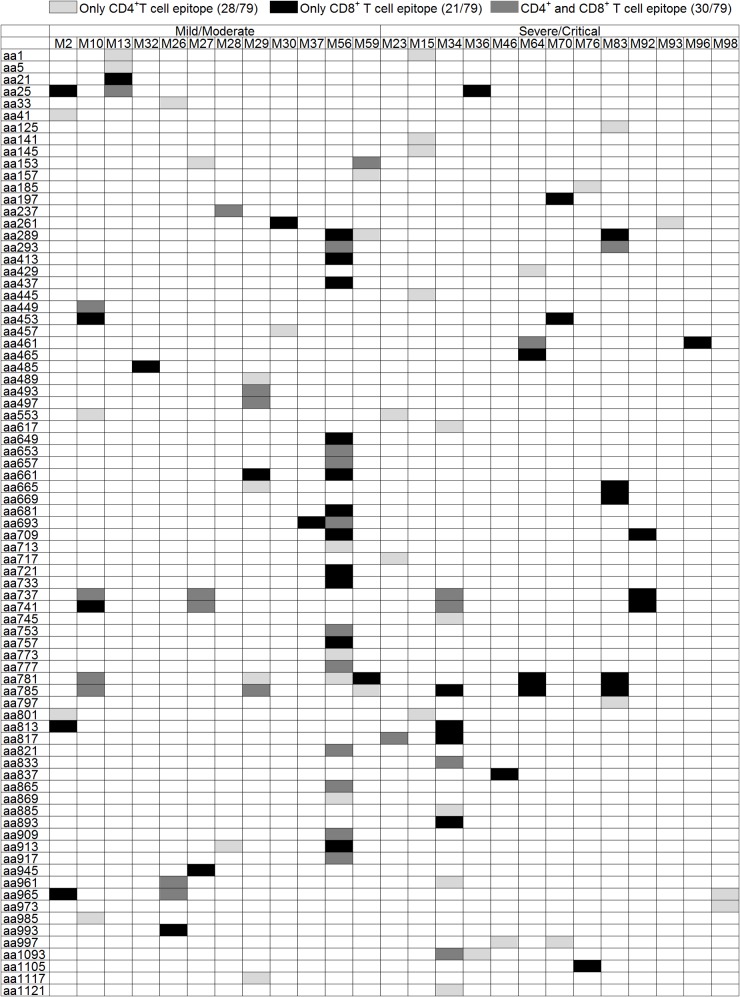
Summary of the T-cell responses to the HTNV-Gn/Gc epitopes in 25 positively responding patients. The column in the table represents the number of each patient (n = 12 for mild/moderate and n = 13 for severe/critical patients). The line of the table represents the start position of 15-mer HTNV-Gn/Gc T-cell epitopes (n = 79) identified in 25 patients. The definition of the epitopes was conducted with the samples from acute stage of each HFRS patient. Light gray boxes indicate positive CD4 responses. Black boxes indicate positive CD8 responses. Dark gray boxes represent positive responses to both CD4^+^ and CD8^+^T cells. Overall, 28 epitopes showed specific responses to CD4^+^T cells, 21 15-mer peptides showed specific responses to CD8^+^T cells and 30 15-mer peptides showed specific responses to both CD4^+^ and CD8^+^T cells. aa, amino acid.

To examine the specific T-cell responses to HTNV-Gn/Gc in HFRS patients, we first screened the T-cell epitopes on HTNV-Gn/Gc. The majority of the participants in this cohort displayed reactive T-cell responses against HTNV-Gn/Gc. Notably, 73.7% (70/95) of the HFRS individuals were responders recognizing at least 1 peptide pool. Among the 70 participants displaying positive responses to HTNV-Gn/Gc, a median of 4 (range 1–11) target peptide pools were detected in each HFRS individual, with a median spot magnitude of 609 (range, 95–4,911) spot-forming cells (SFC)/10^6^ peripheral blood mononuclear cells (PBMCs) against total peptide pools. All peptide pools showed positive responses, among which four peptide pools (P5, P12, P21 and P25) were frequently detected in more than 30% of the subjects, and 7 peptide pools (P17 to P23) contained the most frequently identified HTNV-Gn/Gc reactive T-cell peptides eliciting the strongest responses (S1 Table). Next, we analyzed the positive responses at the single peptide level to identify the T-cell epitopes on HTNV-Gn/Gc. Overall, the single peptide-specific T-cell responses were widely distributed across the primary structure of HTNV-Gn/Gc. Approximately, 155 of the 281 peptides (55.2%) were recognized by at least one person, and among these, 20 peptides were frequently targeted as the immunodominant epitopes in more than 10% of HFRS individuals with diverse HLA backgrounds (Fig 1, Table 1). A median of 6 peptides (range, 1–25) were targeted in a single individual, and the strength of some individual epitope-specific responses reached 1,546 SFC/10^6^ PBMCs. Moreover, to identify a more precise estimation of the epitope-specific T-cell responses, the distribution of *ex vivo* CD4^+^ and CD8^+^T-cell epitopes across the HTNV-Gn/Gc was defined in 25 positive response HFRS patients with sufficient PBMC samples (Fig 2). The detected responses to 28 15-mer peptides were entirely CD4^+^T-cell dependent, indicating that these peptides were CD4^+^T-cell epitopes on HTNV-Gc/Gc. Whereas, the CD8^+^T-cell depletion completely abrogated the interferon (IFN) -γ responses in another 21 15-mer HTNV-Gc/Gc epitopes, confirming CD8^+^T cells as the source of these epitope-specific responses, and these peptides might contain the 9 or 10-mer HTNV-Gc/Gc CTL epitopes. Moreover, we identified 30 additional peptides that could induce both CD4^+^ and CD8^+^T-cell responses against HTNV-Gc/Gc.

**Table 1:** The immunodominant 15-mer T cell epitopes on the glycoprotein of Hantaan virus.

Peptides	Position	Amino acid sequence	Number of positively responding patients	% (/70 patients)	Median of spots/10^6^ PBMC
G6	aa21-35	NVYDMKIECPHTVSF	13	18.57	272
G7	aa25-39	MKIECPHTVSFGENS	11	15.71	271
G37	aa145-159	IVPIHACNMMKSCLI	9	12.86	183
G50	aa197-211	KHGIFDIASVHIVCF	9	12.86	253
G73	aa289-303	IASYSIVGPANAKVP	11	15.71	145
G112	aa445-459	KTLVIGQCIYTITSL	10	14.29	366
G116	aa461-475	SLLPGVAHSIAVELC	8	11.43	304
G117	aa465-479	GVAHSIAVELCVPGF	8	11.43	331
G139	aa553-567	ETYKELKAHGVSCPQ	9	12.86	264
G185	aa737-751	GACTKYEYPWHTAKC	11	15.71	461
G186	aa741-755	KYEYPWHTAKCHYER	12	17.14	388
G196	aa781-795	GLYLDQLKPVGSAYK	11	15.71	156
G197	aa785-799	DQLKPVGSAYKIITI	10	14.29	212
G202	aa805-819	VCVQFGEENLCKIID	9	12.86	431
G205	aa817-831	IIDMNDCFVSRHVKV	9	12.86	102
G208	aa829-843	VKVCIIGTVSKFSQG	8	11.43	93
G209	aa833-847	IIGTVSKFSQGDTLL	8	11.43	95
G210	aa837-851	VSKFSQGDTLLFFGP	9	12.86	134
G241	aa961-975	DFDNLGENPCKIGLQ	7	10.00	147
G242	aa965-979	LGENPCKIGLQTSSI	14	20.00	230

### Comparison of the *ex vivo* T-cell responses against the HTNV glycoprotein in HFRS individuals with different disease severities

**Fig 3:**
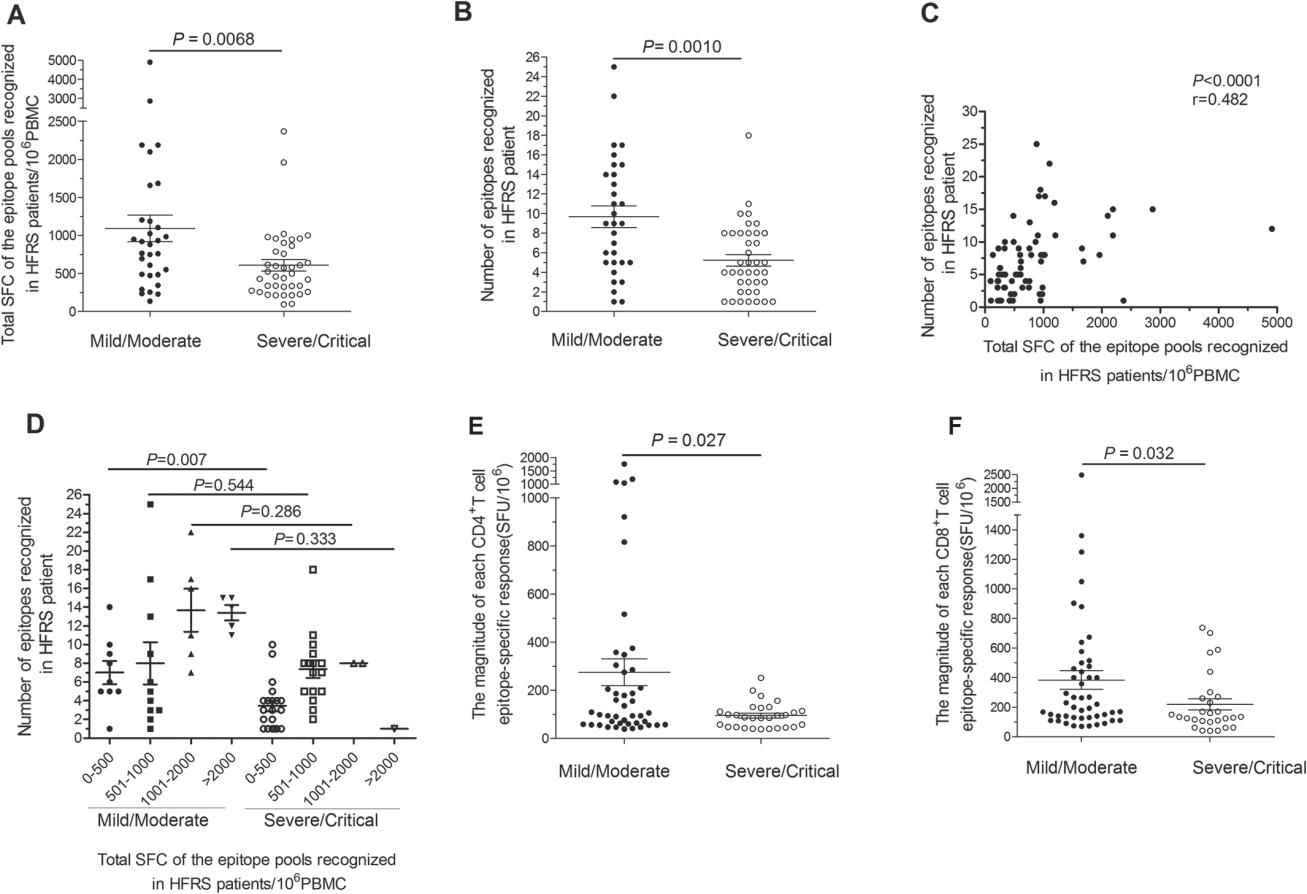
Comparison of antigenic repertoire and magnitude of HTNV-Gn/Gc-specific T-cell responses in patients with different severities. (A-B) Comparison of (A) the total magnitudes (*y axis*) of *ex vivo* ELISPOT IFN-γ T-cell responses to the overlapping peptide pools covering the HTNV-Gn/Gc, and (B) the number of single positive responding HTNV-Gn/Gc 15-mer T cell epitopes (*y axis*) at the acute stage between mild/moderate patients (n = 31) and severe/critical patients (n = 39) (*x-axis*). (C) The correlation between the total magnitude of T-cell responses specific to HTNV-Gn/Gc peptide pools and the number of HTNV-Gn/Gc T-cell epitopes recognized in HFRS patients. (D) Comparison of the recognized epitope number in four subgroups between mild/moderate and severe/critical HFRS patients. The subgroups were divided based on the different magnitude of the specific T-cell responses, including total spot-forming cells (SFC) 0–500, 501–1000, 1001–2000 and more than 2000. Each spot represents a single patient for A-D. (E-F) Comparison of the magnitude of the epitope-specific responses (*y-axis*) of CD4^+^ (E) or CD8^+^ (F) T cells at the acute stage between the two groups in 25 patients (*x-axis*). Each spot represents a single epitope for E-F. The magnitude of the response is represented as the SFC/10^6^ PBMCs. The Wilcoxon rank sum test was used for statistical evaluation.

After the *ex vivo* assessment of the T-cell responses against the HTNV-Gn/Gc, we divided the HFRS patients into two groups according to disease severity and compared their HTNV-Gn/Gc-specific immune responses. Among the 90 patients examined, T-cell responses against HTNV-Gn/Gc were detected in 31 of the 35 (88.6%) patients in the mild/moderate group compared with 39 of the 55 (70.9%) in the severe/critical group (Fisher’s exact chi-square test, *P* = 0.068). Importantly, although the breadth and magnitude of the HTNV-Gn/Gc-specific T-cell responses varied considerably among HFRS individuals, a comparison of the two groups (31 mild/moderate and 39 severe/critical) revealed a total SFC count (the sum of all responses to peptide pools), as an index of the total reactivity against HTNV-Gn/Gc, ranging from 135 to 4,911/10^6^ PBMCs (median 881) for subjects in the mild/moderate group, was significantly higher than that observed for patients in the severe/critical group, with values ranging from 95 to 2,370/10^6^ PBMCs (median 500) (*P* = 0.0068) (Fig 3A). Although the broad distribution of responses was similar in both groups, higher numbers of recognized HTNV-Gn/Gc peptides were observed in the mild/moderate group (mean 10; range 1–25) compared with severe/critical patients, who showed a relatively narrower repertoire directed against a smaller number of HTNV-Gn/Gc epitopes (mean 5; range 1–18) (*P* = 0.0010) (Fig 3B). We next analyzed the correlation between magnitude and breadth of the response specific to HTNV-Gn/Gc and found there was a positive association between the number of epitopes recognized and the total SFC/10^6^ PBMCs of the T-cell responses specific to HTNV-Gn/Gc epitope pools in HFRS patients (*P*<0.0001, r = 0.482) (Fig 3C). Moreover, when dividing the patients into four subgroups according to the total SFC count (/10^6^ PBMCs) including SFC 0–500, SFC 501–1000, SFC 1001–2000 and SFC greater than 2000, the comparison of the recognized epitope number showed a similar tendency that more epitopes are recognized in subjects with mild/moderate disease than that in severe/critical patients in each subgroup, especially in the subgroup with SFC 0–500 (*P* = 0.007) (Fig 3D). Therefore, this higher reactivity, including broader and stronger T-cell responses, was associated with mild rather than severe disease outcomes of HFRS. Consistent with the total T-cell response pattern against HTNV-Gn/Gc, a comparison of the responses targeting CD4^+^ or CD8^+^T-cell epitopes in two groups of 25 HFRS patients (12 mild/moderate and 13 severe/critical) showed similar results. A significant quantitative difference in the magnitude of both CD4^+^ and CD8^+^T-cell responses against each HTNV-Gn/Gc peptide was observed between the two groups. The number of SFC/10^6^ cells was higher in the mild/moderate group (median 106, range 39–1,758 for CD4^+^T-cell responses and median 210, range 70–2,490 for CD8^+^T-cell responses) than that in severe/critical group (median 88, range 39–253 for CD4^+^T-cell responses and median 134, range 40–738 for CD8^+^T-cell responses) (*P* = 0.027 and *P* = 0.032 for CD4^+^ and CD8^+^T-cell responses, respectively) (Fig 3E-3F). In addition, although the difference was not statistically significant, we observed a wider breadth of both CD4^+^ and CD8^+^T-cell epitope responses in the mild/moderate group compared with that in severe/critical patients. To avoid the overlaps between the moderate and severe patients in all above comparisons, we separated the two combined groups into four clinical types as mentioned in Materials and Methods. The comparisons between the mild and critical patients showed similar results with the data between the mild/moderate and severe/critical patients (S1 Fig). The difference in total SFC/10^6^ PBMC of the recognized epitope pools between the two ends of the patients’ spectrum was more marked (*P* = 0.0012) than that between the two combined groups (*P* = 0.0068). Collectively, HTNV-Gn/Gc could elicit robust T-cell immunity with extended epitopic breadth of Gn/Gc specificity. However, in contrast to subjects with mild/moderate HFRS, the responses in patients with severe/critical severity showed narrower antigenic repertoire and much weaker responses against HTNV-Gn/Gc, indicating that HTNV-Gn/Gc-specific T-cell responses would be functional against the HTNV infection during HFRS.

### HTNV exposure results in cytokine production in polyfunctional glycoprotein-specific CD4^+^T cells in HFRS patients

**Fig 4:**
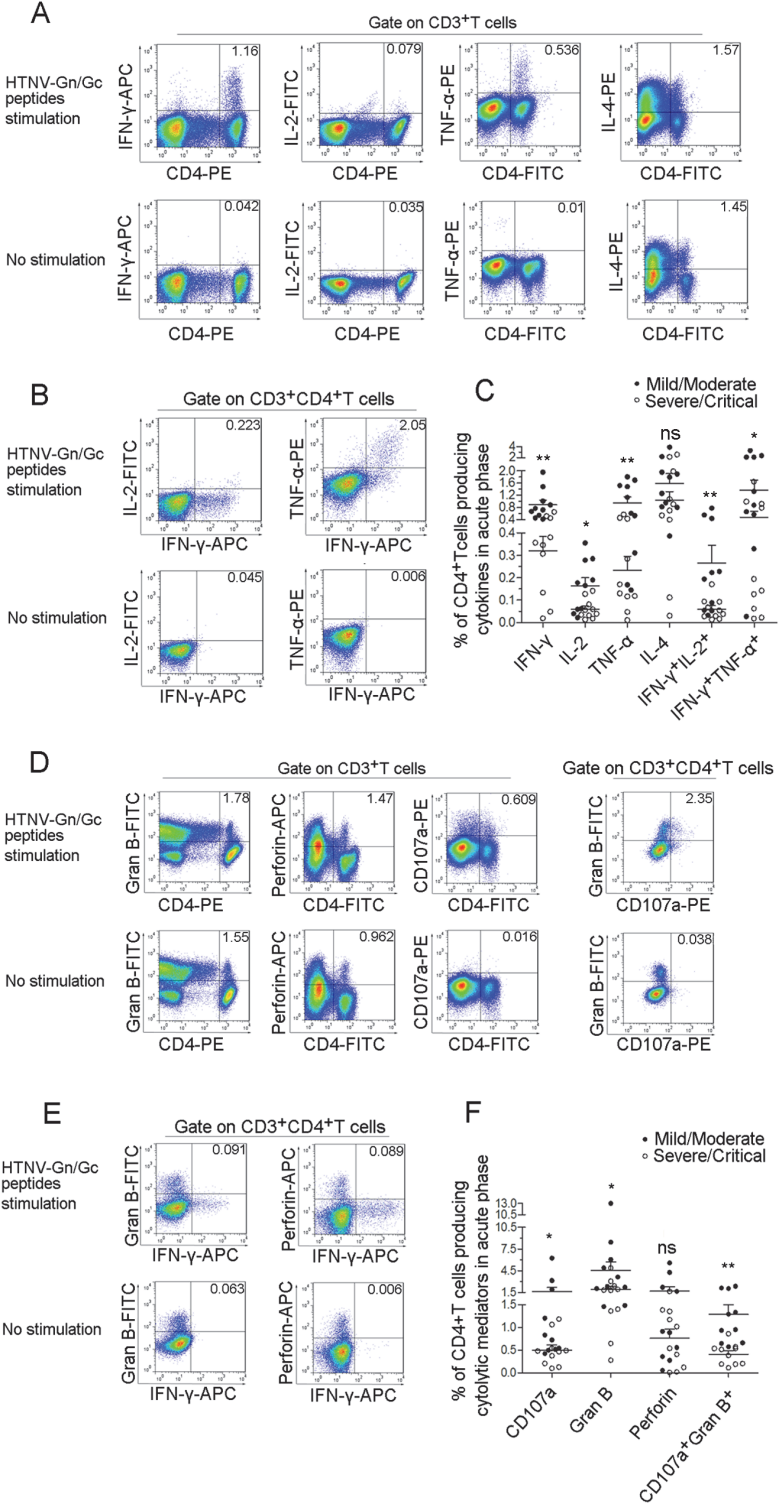
The polyfunctional pattern of cytokine production of HTNV-Gn/Gc-specific CD4^+^T cells. Representative flow cytometric plots of (A) cytokine (IFN-γ, TNF-α, IL-2 and IL-4)-producing CD4^+^T cells in PBMCs during early stage infections within 8 days after disease onset, (B) dual-cytokine production (IFN-γ^+^TNF-α^+^ and IFN-γ^+^IL-2^+^) of HTNV-Gn/Gc-specific CD4^+^T cells, (D) cytotoxic mediator (granzyme B and perforin)-producing and CD107a-expressing CD4^+^T cells in PBMCs and granzyme B^+^CD107a^+^ HTNV-Gn/Gc-specific CD4^+^T cells, (E) IFN-γ^+^granzyme B^+^ and IFN-γ^+^perforin^+^ cells of the HTNV-Gn/Gc-specific CD4^+^T cells. FACS contour plots were gated on CD3^+^ cells for the analysis of single intracellular cytokine and gated on CD3^+^CD4^+^ T cells for the analysis of dual intracellular-cytokine (percentages of double positive cells are shown) frequency. The *Upper* lane in (A), (B), (D) and (E) shows the results after HTNV-Gn/Gc peptides stimulation, and the *lower* lane shows the results without peptide stimulation. The numbers denote the percentage of cells within the boxed regions. Comparison of the frequencies of (C) single cytokine (IFN-γ, TNF-α, IL-2 or IL-4) and dual-cytokine (IFN-γ^+^TNF-α^+^ or IFN-γ^+^IL-2^+^)-producing CD4^+^T cells, (F) the cytotoxic mediators (granzyme B and perforin) and the expression of CD107a or granzyme B^+^CD107a^+^ cells of the HTNV-Gn/Gc-specific CD4^+^T cells between the two groups with different severities. Each dark spot in (C) and (F) represents a single mild/moderate patient and each circle represents a single severe/critical individual. The Wilcoxon rank sum test was used for statistical evaluation; *P<0.05, **P<0.01, ns, not significant. Gran B, granzyme B.

Given the importance of specific T-cell responses against the HTNV-Gn/Gc in controlling HTNV infection, we further investigated the cytokine secretion in T cells from HFRS patients after HTNV-Gn/Gc stimulation. At the acute phase of HFRS, HTNV-Gn/Gc-specific CD4^+^T cells displayed increased cytokine production, predominantly characterized by the Th1 cytokine profile upon recognition of the peptides. IFN-γ was the primary cytokine produced (median 0.486%, range 0.019%-1.950% of total CD4^+^T cells). A high frequency of tumor necrosis factor (TNF)-α-producing cells was also observed (median 0.372%, range 0.011%-1.800% of total CD4^+^T cells). A few cells produced interleukin (IL)-2 (median 0.061%, range 0.013%-0.355% of total CD4^+^T cells), whereas virtually IL-4-producing cells could also be detected (median 1.011%, range 0.031%-3.870% of total CD4^+^T cells) (Figs 4A, S2, S3). Further analysis revealed dual-cytokine production in HTNV-Gn/Gc-specific CD4^+^T cells. Subsets of IFN-γ-producing CD4^+^T cells simultaneously secreted IL-2 (median 0.079% of total CD4^+^T cells) or TNF-α (median 0.723% of total CD4^+^T cells), indicating that specific CD4^+^T cells induced a range of polyfunctional responses (T cells making at least 2 cytokines simultaneously) against HTNV-Gn/Gc stimulation (Figs 4B, S2, S3). Next, we compared the cytokine secretion levels in patients with different HFRS severities. Overall, the functions of the specific CD4^+^T cells were almost impaired in severe/critical patients, while stronger CD4^+^T-cell responses were observed in patients with milder HFRS. HTNV-Gn/Gc peptides elicited substantially higher frequencies of single IFN-γ (median 0.746% of total CD4^+^T cells), IL-2 (median 0.172% of total CD4^+^T cells) and TNF-α (median 0.868% of total CD4^+^T cells) or dual-cytokine (median 0.209% for IFN-γ^+^IL-2^+^CD4^+^T cells and median 1.100% for IFN-γ^+^TNF-α^+^CD4^+^T cells) secretion from specific CD4^+^T cells in mild/ moderate HFRS patients. In contrast, patients with severe/critical HFRS showed less single cytokine IFN-γ (median 0.351% of total CD4^+^T cells, *P*<0.001), IL-2 (median 0.048% of total CD4^+^T cells, *P* = 0.023) and TNF-α (median 0.150% of total CD4^+^T cells, *P* = 0.005) or dual-cytokine (median 0.047% for IFN-γ^+^IL-2^+^CD4^+^T cells, *P* = 0.015 and median 0.169% for IFN-γ^+^TNF-α^+^CD4^+^T cells, *P* = 0.049) production. No significant differences in IL-4 production were detected between patients with different severities of infection (Fig 4C).

Notably, some CD4^+^T cells displayed adequate cytolytic capacity and appeared to be highly functional, secreting cytolytic effectors, including granzyme B (median 2.120%, range 0.275%-13.000% of total CD4^+^T cells) and perforin (median 0.899%, range 0.005%-5.560% of total CD4^+^T cells); upregulated CD107a expression (median 0.527%, range 0.098%-6.230% of total CD4^+^T cells) was observed after recognition of the HTNV-Gn/Gc peptides (Figs 4D, S4, S5). The simultaneous production of granzyme B and CD107a expression (median 0.608% of total CD4^+^T cells) was also observed in subsets of CD4^+^T cells (Figs 4D, S4, S5). This important finding is consistent with several previous studies, showing the cytolytic capacity of virus-specific CD4^+^T cells, defined as a new “ThGzmB^+^ cell” subset of CD4^+^T cells. Although the rapid production of IFN-γ or granules by T cells is important in defense against virus infections, there seems to be few double IFN-γ^+^granzyme B^+^ or IFN-γ^+^perforin^+^ cells were visualized in each CD4^+^T-cell population analyzed (Fig 4E). Similarly, obvious higher frequency of granzyme B or CD107a expression was observed in the CD4^+^T cells from mild/moderate patients (median 2.965% for granzyme B and 0.777% for CD107a of total CD4^+^T cells) compared with severe/critical patients (median 1.795% for granzyme B and 0.469% for CD107a of total CD4^+^T cells) (*P* = 0.035 for granzyme B and *P* = 0.015 for CD107a). We also observed a trend towards higher levels of perforin^+^CD4^+^T cells in the mild/moderate group although this difference was not significant (*P* = 0.353) (Fig 4F).

### The Th1 and ThGzmB^+^ cell responses against HTNV glycoprotein are associated with the CTL response and level of viremia in HFRS patients

**Fig 5:**
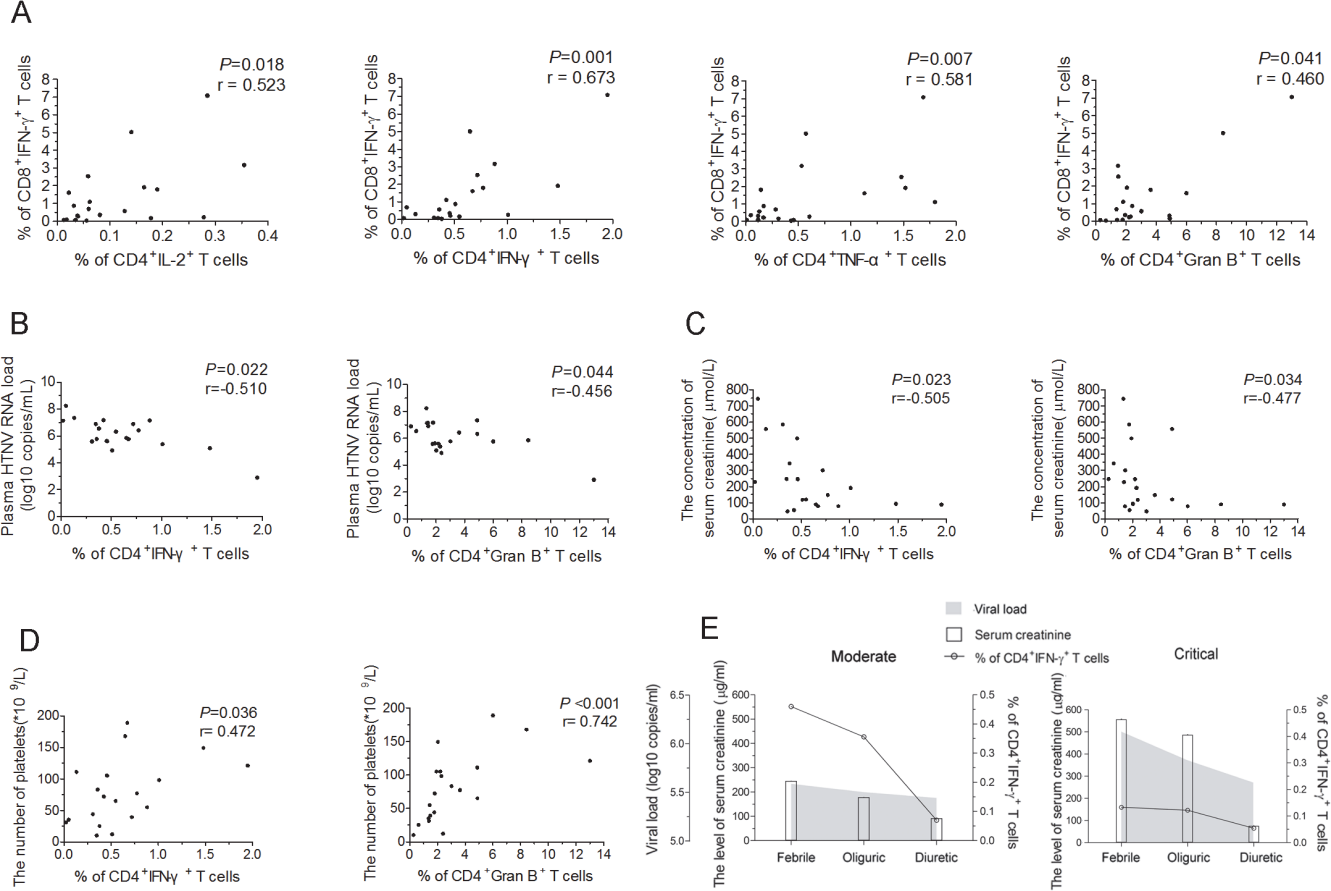
The correlations of HTNV-Gn/Gc-specific CD4^+^T-cell responses with CD8^+^T-cell immunity, viremia and clinical parameters. (A) Analysis ofthe correlations between the frequency of CD4^+^IL-2^+^T cells, CD4^+^IFN-γ^+^T cells, CD4^+^TNF-α^+^T cells, or CD4^+^granzyme B^+^T cells (*x axis*) and the percentage of CD8^+^IFN-γ^+^T cells (*y axis*) during the acute stage of HFRS. (B-D) Analysis of the relationship between the percentage of IFN-γ or granzyme B-producing CD4^+^T cells and the plasma HTNV RNA load (B), the serum creatinine levels (C), or platelets numbers (D) during acute HFRS. Each spot represents a single patient. (E) Longitudinal assessment of the HTNV RNA load, serum creatinine and the frequency of HTNV-Gn/Gc-specific IFN-^+^CD4^+^T cells in representative patients with moderate severity and representative patients with critical severity during the course of the disease. The line indicates the level of IFN-^+^CD4^+^T cells, white bars indicate the serum creatinine levels, and the shaded areas show the plasma HTNV RNA load of the patients tested. The Spearman’s rank test was used for statistical evaluation. Gran B, granzyme B.

Next, we measured the relationships between the frequencies of IFN-γ^+^CTLs and each Th1 cytokine-secreting CD4^+^T-cell subset. As expected, the frequencies of IL-2^+^CD4^+^, IFN-γ^+^CD4^+^ or TNF-α^+^CD4^+^T-cell subsets were positively correlated with the frequency of IFN-γ^+^CTLs (*P* = 0.018, r = 0.523 for IL-2^+^CD4^+^T cells; *P* = 0.001, r = 0.673 for IFN-γ^+^CD4^+^T cells; *P* = 0.007, r = 0.581 for TNF-α^+^CD4^+^T cells) (Fig 5A). In addition, the frequency of the granzyme B^+^CD4^+^T cells was also positively associated with the frequency of IFN-γ^+^CTLs (*P* = 0.041, r = 0.460) (Fig 5A), suggesting that ThGzmB^+^ cells are potential cytolytic effector cell subsets against HTNV infection.

In a previous study, we showed that upon HTNV infection, the direct onset of viremia was observed and higher levels of HTNV RNA load in the plasma of the HFRS patients was obviously correlated with much more severe HFRS, suggesting that the plasma HTNV RNA load could be used as a prognostic marker and predictor of the disease severity in HFRS [34]. The further analysis revealed highly significant associations between the higher frequencies of IFN-γ^+^CD4^+^T cells or granzyme B^+^CD4^+^T cells and lower HTNV RNA load in the acute phase of the HFRS (*P* = 0.022, r = −0.510 for IFN-γ^+^CD4^+^T cells and *P* = 0.044, r = −0.456 for granzyme B^+^CD4^+^T cells) (Fig 5B), indicating that higher levels of activated CD4^+^T cells against HTNV-Gn/Gc during the initial phase of HFRS mediate the better control of HTNV viremia. In addition, because the increased capillary permeability of the acute renal injury and thrombocytopenia are hallmarks in the course of HFRS, the serum creatinine levels and the platelet counts in HFRS patients typically provide a better prediction of disease severity [35]. Notably, we observed inverse correlations between the frequency of IFN-γ or granzyme B-secreting CD4^+^T cells and the level of serum creatinine (*P* = 0.023, r = −0.505 for IFN-γ^+^CD4^+^T cells and *P* = 0.034, r = −0.477 for granzyme B^+^CD4^+^T cells) (Fig 5C), while positive correlations between the frequency of IFN-γ or granzyme B-secreting CD4^+^T cells and the number of platelets in HFRS patients (*P* = 0.036, r = 0.472 for IFN-γ^+^CD4^+^T cells and *P*<0.001, r = 0.742 for granzyme B^+^CD4^+^T cells) (Fig 5D).

To better assess the effect of viral control through HTNV-Gn/Gc-specific CD4^+^T cells in HFRS patients, we carefully examined the kinetics of the frequency of IFN-γ-secreting CD4^+^T cells, the plasma HTNV RNA load, and the serum creatinine levels during the course of HFRS in patients with different disease severities. Consistent with previous studies, a rapid decrease in the plasma viral load from the febrile to convalescence stage was observed in both mild/moderate and severe/critical patients. This trend was coincident with a reduction in the frequency of IFN-γ secreting CD4^+^T cells and the levels of serum creatinine (Fig 5E). However, the levels of these three parameters were distinct at the febrile stage in the two HFRS groups. In mild/moderate patients, the frequency of IFN-γ-secreting CD4^+^T cells was obviously higher than that in severe/critical patients, whereas both the plasma viral load and serum creatinine levels in mild/moderate patients were lower compared with those in severe/critical patients at the initial stage of HFRS (Fig 5E). This observation primarily reflects the potentially function of antiviral effectors of HTNV-Gn/Gc-specific CD4^+^T cells at the acute stage of the disease, which might be a key determinant of the level of virus replication and the extent of renal injury in HFRS. This finding was further highlighted in the analysis of the viral control of CD4^+^T cells against HTNV infection.

### The cytotoxic capacity of granzyme B-secreting HTNV glycoprotein-specific CD4^+^T cells correlated with disease severity

**Fig 6:**
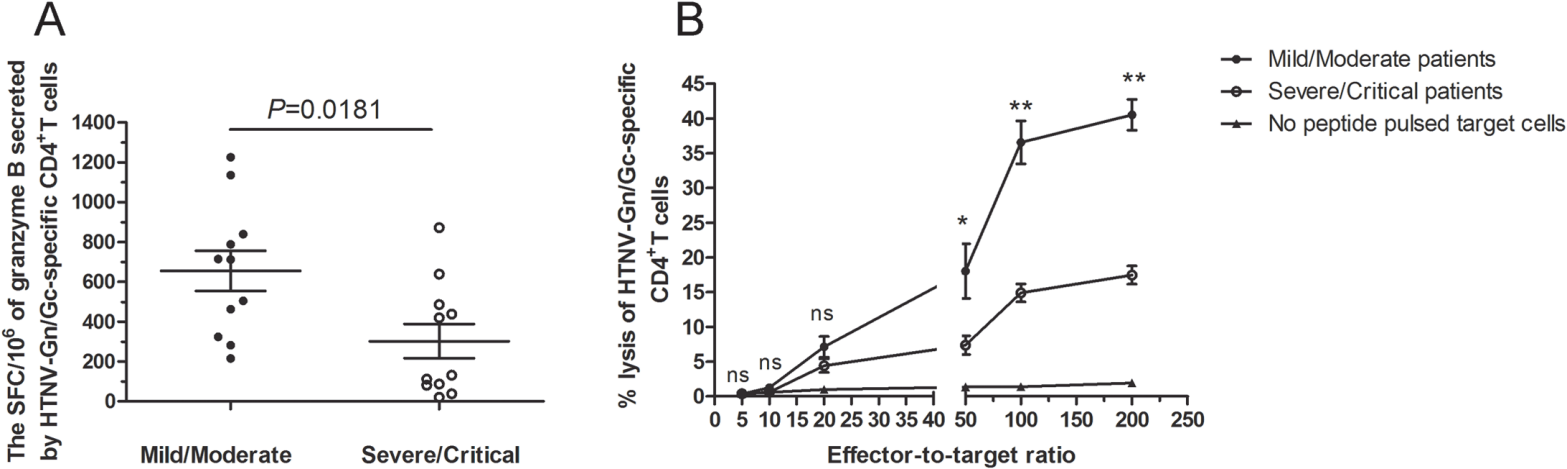
The magnitude of granzyme B production and cytotoxic capacity of HTNV-Gn/Gc-specific CD4^+^T cells. (A) Comparison of the SFC/10^6^ cells of granzyme B secreted by CD4^+^T cells specific to HTNV-Gn/Gc in ELISPOT assay between mild/moderate patients and severe/critical individuals in acute phase of HFRS. (B) The kinetics of specific lysis of peptides-pulsed target cells by the HTNV-Gn/Gc-specific CD4^+^T-cell population assessed by in vitro cell-mediated cytotoxicity assay. The CD4^+^T cells isolated from the PBMCs of the HFRS patients were used as effector cells, and the Epstein Barr Virus (EBV) transformed autologous B lymphoblastic cell line (B-LCL) or MHC class Ⅱ partial matched B-LCL of each patient pulsed with HTNV-Gn/Gc peptides were used as target cells. The effector-to-target ratios included 200:1, 100:1, 50:1, 20:1, 10:1 and 5:1. The dark spots and the circles represent the mean lysis percentage of CD4^+^T cells from mild/moderate and severe/critical patients, respectively to kill the HTNV-Gn/Gc peptides-pulsed target cells. The triangles represent the mean lysis percentage of CD4^+^T cells from all the patients to kill no peptide-pulsed target cells. The comparison of lysis percentages between the mild/moderate and severe/critical groups at each effector-to-target ratio were showed as *P<0.05, **P<0.01, and ns, not significant. The Wilcoxon rank sum test was used for statistical evaluation.

We next set out to determine whether the granzyme B-secreting CD4^+^T cells could kill the HTNV-Gn/Gc peptide pools-pulsed target cells. Firstly, *ex vivo* granzyme B enzyme-linked immunospot (ELISPOT) assay showed that the HTNV-Gn/Gc epitope peptide pools could stimulate CD4^+^T cells of HFRS patients to produce granzyme B with the average magnitude of 450 SFC/10^6^ cells (range, 21–1,226) (S6 Fig, n = 11 for mild/moderate and severe/critical patients, respectively). The comparison between the two groups showed that the magnitude of granzyme B-producing CD4^+^T-cell response was stronger in mild/moderate patients than that in severe/critical individuals (median 712, range 215–1,226 SFC/10^6^ cells for mild/moderate group and median 131, range 21–872 SFC/10^6^ cells for severe/critical group, *P* = 0.0181) (Fig 6A). Second, the biological cytolytic function of CD4^+^T cells was determined by the cytotoxic assay, in which the CD4^+^T cells of HFRS patients could lyse HTNV-Gn/Gc peptides-pulsed autologous or MHC class Ⅱ partial matched B lymphoblastic cell lines (B-LCLs) with the average percentage of 22.63 (range, 10.92–45.93) at effector-to-target cell ratio of 200:1. Moreover, we also observed that the percentages of cytotoxicity in mild/moderate group seems to be higher than that in severe/critical group at effector-to-target cell ratio of 200:1(*P* = 0.0002), 100:1(*P* = 0.0002) and 50:1 (*P* = 0.0229) (Fig 6B). The cytotoxic capacity of each patient was showed in S7 Fig (n = 7 for mild/moderate and n = 9 for severe/critical patients). Collectively, these results suggested that ThGzmB^+^ cell subset might participate in the immune response against HTNV infection through the production of granzyme B and cell-mediated cytolytic function during the early stages of the disease, defining a specific CD4^+^T cell functional phenotype for HTNV control.

### *Ex vivo* CD4^+^T-cell expansion against HTNV glycoprotein during the acute phase predict the severity of HFRS

**Fig 7:**
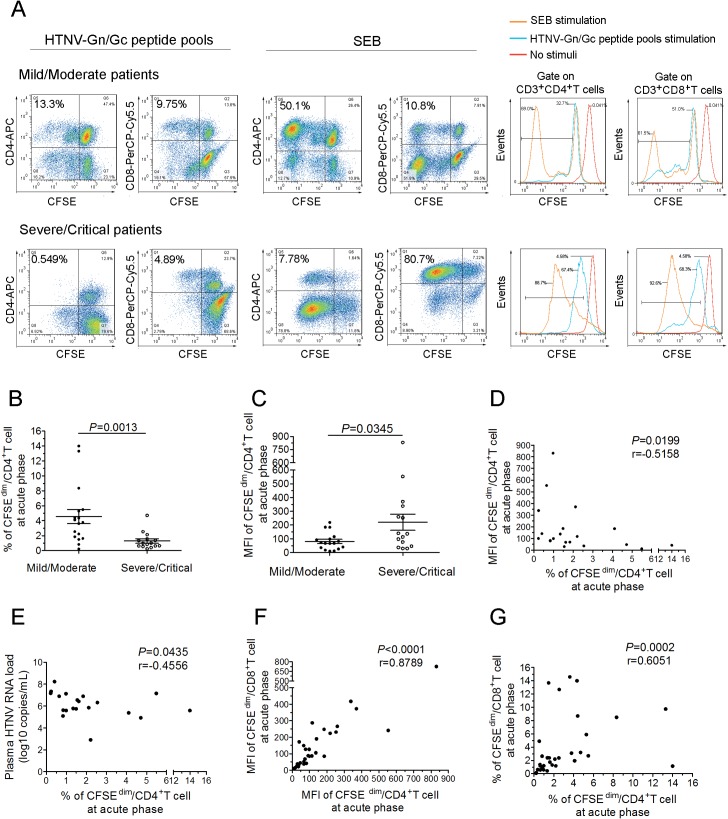
The expansion capacity of HTNV-Gn/Gc-specific CD4^+^T cells is associated with viremia control in HFRS patients. (A) Representative flow cytometric plots of the expansion percentage of CD4^+^ or CD8^+^T cells stimulated by the HTNV-Gn/Gc or polyclonal activator SEB control during acute HFRS. The expansion extent of the HTNV-Gn/Gc-specific CD4^+^ or CD8^+^T cells is shown in the *upper left* quadrants of each figure, reflecting the decrease of CFSE in the dividing CD4^+^ or CD8^+^T cells. The numbers denote the percentage of cells within the boxed regions. The overlay of the three conditions in histograms clearly demonstrate that the CFSE curve of both CD4^+^ and CD8^+^T cells from the SEB stimulation is shifted more to the right than that stimulated by the HTNV-Gn/Gc. The *upper* and the *lower lanes* show the expansion capacities of the representative mild/moderate and severe/critical patient, respectively. (B-C) Comparison of the percentage (B) and MFI (C) of CFSE^dim^ CD4^+^T cells stimulated by HTNV-Gn/Gc at the acute phase of HFRS between mild/moderate and severe/critical patients. (D) Analysis of the correlation between the percentage and the MFI of CFSE^dim^ CD4^+^T cells. (E) Analysis of the correlations between the percentage of CFSE^dim^ CD4^+^T cells and the plasma HTNV RNA load of HFRS. (F-G) The correlation of the MFI (F) or percentage (G) between CFSE^dim^ CD4^+^T cells and CFSE^dim^ CD8^+^T cells stimulated by HTNV-Gn/Gc during acute HFRS. Each spot represents a single patient. The Wilcoxon rank sum test and Spearman’s rank test were used for statistical evaluation. SEB, staphylococcal enterotoxin B; MFI, mean fluorescence intensity; CFSE, carboxyfluorescein succinimidyl ester.

We next assessed whether the antivirus effect of CD4^+^T cells was dependent on the capacity of expansion. The CFSE staining assay showed that HTNV-Gn/Gc-specific CD4^+^T-cell expansion was readily detectable in HFRS individuals at the acute phase of the disease (representative in Fig 7A and others in S8 and S9 Figs). However, a significant difference in the CD4^+^T-cell expansion to HTNV-Gn/Gc between two different groups was observed. The percentage of CFSE^dim^ CD4^+^T cells in mild/moderate patients was higher than that in severe/critical patients (Fig 7B, *P* = 0.0013), and the mean fluorescence intensity (MFI) of CFSE^dim^ CD4^+^T cells was lower in milder patients than that in more severe HFRS individuals (Fig 7C, *P* = 0.0345). Meanwhile, the MFI of CFSE^dim^ CD4^+^T cells was inversely associated with the percentage of CFSE^dim^ CD4^+^T cells at acute phase of HFRS (Fig 7D, *P* = 0.0199, r = −0.5158). These data indicated that milder disease severity was associated with more vigorous HTNV-Gn/Gc-specific CD4^+^T-cell expansion, whereas an impaired expansion capacity of HTNV-Gn/Gc-specific CD4^+^T cells was shown in severe/critical patients. However, when stimulated with the polyclonal activator SBE as control, both CD4^+^ and CD8^+^ T cells of the HFRS patients presented proliferative activity with a large proportion of CFSE^dim^ cells (representative in Fig 7A and others in S9 Fig), which was obviously higher than that stimulated with HTNV-Gn/Gc peptides (S10 Fig, *P* = 0.0049 for CD4^+^T cell and *P* = 0.0005 for CD8^+^T cell), suggesting that the impaired T-cell expansion in severe/critical HFRS patients may be refractory to the specific HTNV-Gn/Gc stimulation, but not susceptible to a systemic cell death after activation.

Because the effective cytokine-secreting CD4^+^T-cell response plays an important role in the control of HTNV viremia, we reasoned that the *ex vivo* CD4^+^T-cell expansion should also correlate with the decreased HTNV RNA load. During the acute phase of the disease, the percentage of CFSE^dim^ CD4^+^T cells showed a significant inverse correlation with the maximum plasma viral load in each HFRS patient (Fig 7E; *P* = 0.0435, r = −0.4556), suggesting that the acquisition of proliferative potential and capacity might be an essential character for specific CD4^+^T cells in protection from HTNV infection. Previous studies have demonstrated a strong linkage between HTNV-specific CD8^+^T-cell expansion and the control of HTNV infection [18]. Thus, when we analyzed the relationship of the proliferation between HTNV-Gn/Gc-specific CD4^+^ and CD8^+^T cells, both the percentage and the MFI of the CFSE^dim^ cells showed an expected positive correlations between CD4^+^ and CD8^+^T cells at the initial phase of infection in HFRS patients (Fig 7F-7G; *P*<0.0001, r = 0.8789 for MFI and *P* = 0.0002, r = 0.6051 for percentage). In fact, when we comparing the CD8^+^T cells for the difference of proliferation capacity in the two groups, we found that, similar with CD4^+^T cells, both the MFI and the percentage of CFSE^dim^ CD8^+^T cells were higher in milder patients than that in more severe individuals (S11 Fig, *P* = 0.0087 for percentage and *P* = 0.0454 for MFI). Taken together, these data demonstrate a significant expansion of HTNV-Gn/Gc specific CD4^+^T cells during the acute phase of HFRS, associated with the control of HTNV viremia in patients showing a milder degree of disease severity.

### The phenotypic properties of effective HTNV glycoprotein-specific CD4^+^T cells in peripheral blood of HFRS patients

**Fig 8:**
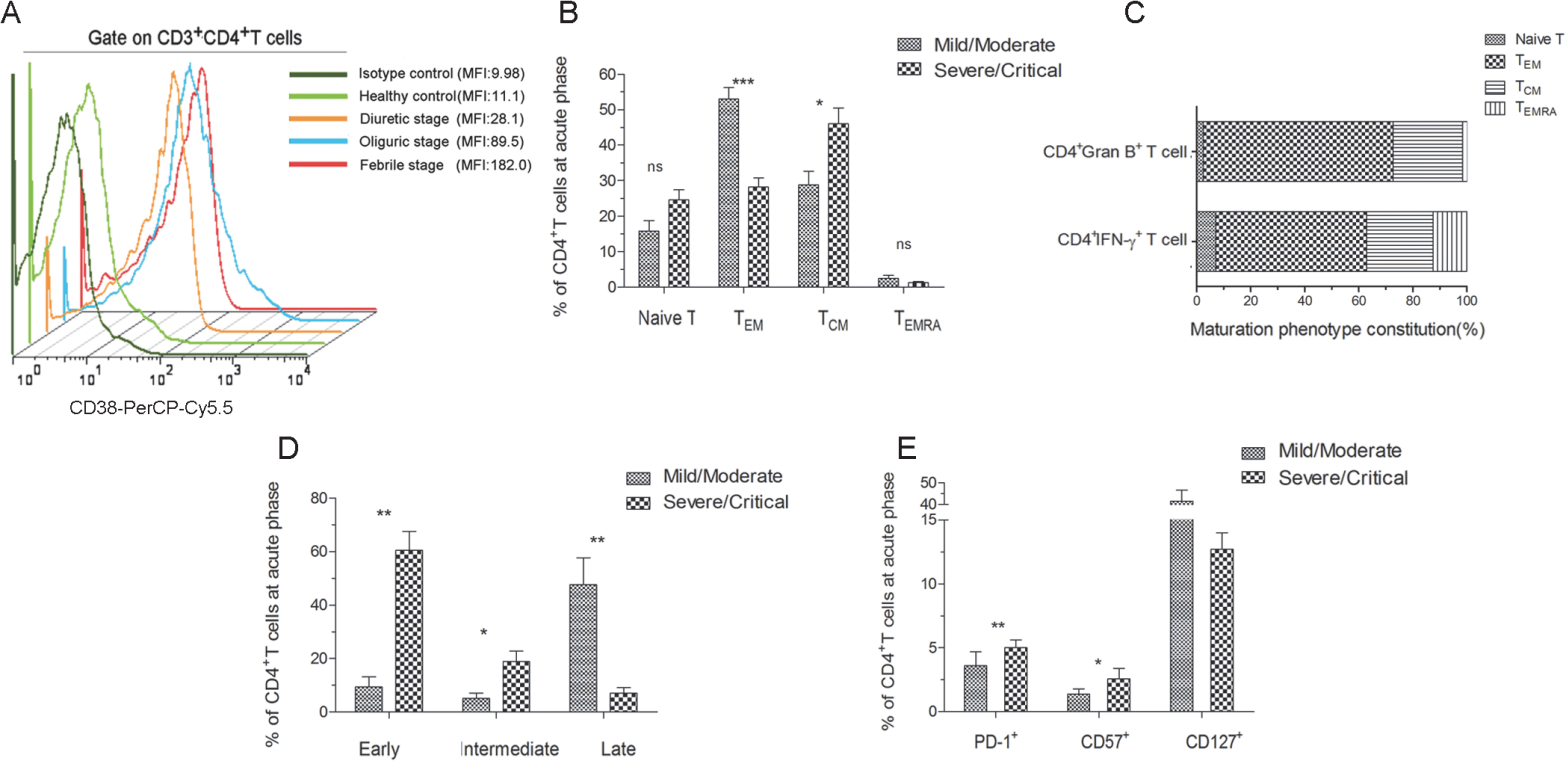
Analysis of the active, memory or differentiation phenotypes of HTNV-Gn/Gc-specific CD4^+^T cells in HFRS patients. (A) Representative histogram of CD38 expression on HTNV-Gn/Gc-specific CD4^+^T cells during different stages of the disease. FACS contour plots were gated on CD3^+^CD4^+^T cells. (B) Comparison of the memory phenotype constitution percentage of naïve (CCR7^+^CD45RA^+^), effector memory (CCR7^–^CD45RA^–^), central memory (CCR7^+^CD45RA^–^) and transitional effector memory (CCR7^–^CD45RA^+^) CD4^+^T-cell subsets in mild/moderate patients and severe/critical patients during acute HFRS. (C) The memory phenotype constitution percentage in IFN-γ or granzyme B-producing CD4^+^T cells. (D) Comparison of the differentiation phenotype percentage of early (CD27^+^CD28^+^), intermediate (CD27^–^CD28^+^) and full differentiation (CD27^–^CD28^–^) CD4^+^T-cell subsets in mild/moderate patients and severe/critical patients at acute stage of HFRS. (E) Comparison of the PD-1, CD57 and CD127 expression on CD4^+^T cells in mild/moderate and severe/critical patients during acute phase of HFRS. The Wilcoxon rank sum test was used for statistical evaluation; *P<0.05, **P<0.01, *** *P*<0.001, ns, not significant. MFI, mean fluorescence intensity; Gran B, granzyme B.

To characterize the overall phenotype of HTNV-Gn/Gc-specific CD4^+^T cells, we first defined the activation extent of CD4^+^T cells. After stimulation with HTNV-Gn/Gc-overlapping peptide pools, the CD38 expression in circulating blood CD4^+^T cells was upregulated, peaking at the febrile stage of HFRS (MFI 182) (Fig 8A). The maximal activity of HTNV-Gn/Gc-specific CD4^+^T cells at the febrile stage might reflect a rapid and prominent CD4^+^T-cell response against HTNV infection after disease onset, prompting further examination of the phenotype characteristics of efficient CD4^+^T cells.

We next determined the memory phenotype of HTNV-Gn/Gc-specific CD4^+^T cells in HFRS patients. To this end, the expression of CCR7, CD45RA and CD127 in responding CD4^+^T cells was evaluated in patients with different severities. Effector memory (T_EM_) CD4^+^T cells (CCR7^–^CD45RA^–^) (median 49.2% of CD4^+^T cells) are typically observed in patients with mild/moderate HFRS, while the individuals with severe/critical disease were enriched for central memory (T_CM_) CD4^+^T cells (CCR7^+^CD45RA^–^) (median 50.2% of CD4^+^T cells) (Fig 8B). There was no observable difference between the two groups in the levels of naïve (CCR7^+^CD45RA^+^) or transitional (CCR7^–^CD45RA^+^) T cell populations. An analysis of the formulation of the memory phenotype in responsive IFN-γ or granzyme B-secreting CD4^+^T cells for the two patient samples revealed CCR7^–^CD45RA^–^CD4^+^T_EM_ cells as the predominant T cell subset (55.9% T_EM_ in IFN-γ^+^CD4^+^T cells and 70.2% T_EM_ in granzyme B^+^CD4^+^T cells) (Fig 8C). We also observed an abundant percentage of CD127^high^ CD4^+^T cells in mild/moderate patients, and these cell numbers were higher than those detected in severe/critical HFRS individuals (Fig 8E). Thus, these findings suggested that HTNV-Gn/Gc-specific CD4^+^T cells exhibiting a CCR7^–^CD45RA^–^CD127^high^ effector memory phenotype might contribute to the milder severity of HFRS.

Next, these cells were examined for the expression of markers associated with T cell differentiation and maturation. Fig 8D illustrates the CD4^+^ differentiation populations delineated according to the coexpression of CD27 and CD28 maturation markers in the peripheral blood of HFRS patients. Although various proportions of differentiated subsets were observed within the CD4^+^T-cell population in HFRS patients, the CD27^–^CD28^—^highly differentiation subset constituted the majority of the CD4^+^T-cell population in mild/moderate patients (median 48.30% of CD4^+^T cells), while these cells were only a minor component of the HTNV-Gn/Gc-specific CD4^+^T cells in severe/critical patients (median 6.94% of CD4^+^T cells, *P* = 0.004). In contrast, patients with severe/critical HFRS exhibited a CD27^+^CD28^+^ early differentiation phenotype (median 64.50% of CD4^+^T cells), and this effect was much higher than that in mild/moderate patients (median 7.06% of CD4^+^T cells, *P* = 0.004). Thus, we speculated that patients exhibiting an initial differentiated phenotype of HTNV-Gn/Gc-specific CD4^+^T cells during the acute phase of the disease would be exposed to higher levels of HTNV replication, resulting in more severe HFRS. In contrast, the CCR7^—^and CD28^–^CD27^—^phenotypes could represent fully differentiated T_EM_ CD4^+^T cells in mild/moderate HFRS patients, potentially contributing to the effective control of HTNV infections.

Moreover, we analyzed the expression of inhibitory receptors on HTNV-Gn/Gc-specific CD4^+^T cells, potentially representing another factor involved in CD4^+^T cell function against HTNV infection. To this end, we assessed the expression of programmed cell death ligand 1 (PD-1) and CD57 in responding CD4^+^T cells during acute HTNV infection. A substantial fraction of CD4^+^T cells expressed both PD-1 and CD57 early after symptom onset in the HFRS patients. As shown in Fig 8E, patients with severe/critical HFRS exhibited a significant PD-1^high^ CD57^high^ phenotype (median 5.63% for PD-1 and 2.14% for CD57 in CD4^+^T cells) compared with the PD-1^low^ CD57^low^ phenotype observed in mild/moderate patients (median 1.36% for PD-1 and 0.44% for CD57 in CD4^+^T cells) (*P* = 0.004 for PD-1 and *P* = 0.032 for CD57), suggesting that the increased expression of inhibitory receptors might be associated with CD4^+^T-cell dysfunction or downregulation of the effector CD4^+^T-cell response against HTNV infection, leading to a more serious degree of HFRS.

## Discussion

The host immune response is crucial for the control of viral infection through orchestrated activities of different components of the immune system, among which, cellular immune response greatly contributes to the outcome of virus infectious diseases. Studies of the cellular immunological features of pathogenic HTNV infection provide insight into the understanding of HFRS pathogenesis in humans. In this study, we provided, for the first time, that HTNV could induce both Th1 and ThGzmB^+^ cell responses involving in the defense against HTNV infection; we showed that HTNV-Gn/Gc-specific CD4^+^T-cell immunity with broad reactivity, polyfunctionality, expansion capacity, and effector phenotype, inversely correlated with plasma viral load and the HFRS disease outcome; we further provided evidence that HTNV-Gn/Gc-specific CD4^+^T cells mediated host defense against HTNV infection maybe through the Th1 induced antiviral condition of the host cells and the cytotoxic effect of ThGzmB^+^ cells.

As only a few CTL epitopes have been defined on HTNV-Gn/Gc in HFRS patients, T-cell epitope-based preventive HTNV vaccine studies could only benefit from a focus on previously defined CTL epitopes on HTNV-NP. This limitation prompted a search for novel protective T-cell epitopes on HTNV. In the present study, we first performed the systematic identification of CD4^+^ and CD8^+^T-cell epitopes on HTNV-Gn/Gc. The immunodominant regions on these novel immune epitopes were subsequently analyzed and defined based on frequent observation in a number of HFRS individuals of diverse HLA backgrounds. Notably, immunodominance was precisely defined as the most frequently recognized epitope in a cohort of patients, with a strong magnitude of response relative to the total response. Although these experiments did not provide evidence that dominant HTNV-Gn/Gc-specific T cells kill or suppress virus-infected cells more efficiently than subdominant T cells, the immunodominant region and epitopes might merit special attention for the potential development of a novel vaccines that would generate or boost T-cell responses against HTNV in humans.

Previous studies have suggested that NP are major targets of HTNV-specific CTL recognition in humans [14–15,17,23,36–37]. The present *ex vivo* study verified that Gn/Gc, as another immunogenic protein of HTNV, could elicit specific CD4^+^ and CD8^+^ effector T-cell responses and provide obvious protection against HTNV infection. Importantly, HTNV-Gn/Gc-specific T-cell responses were not detected in 26.3% of HFRS patients. This result potentially reflects a response level below the threshold of assay detection or protection through other immune responses, such as HTNV-specific neutralizing antibody [13,38]. In the 73.7% responding patients, a considerable variation in HTNV-Gn/Gc-specific T-cell epitope recognition patterns was observed between different individuals, including the magnitude and breadth of the responses. Indeed, functional differences in specific T-cell responses for different HTNV-Gn/Gc epitopes were observed, particularly because patients with mild/moderate HFRS always showed stronger T-cell responses compared with severe/critical patients. Moreover, we also observed a general wider breadth of T-cell epitope responses in the mild/moderate group compared with that in severe/critical patients, although there is no statistical difference in the number of recognized epitopes between milder and more severe patients in some subgroups with different total SFC, which may be due to the small sample sizes after subdivision in each subgroup. The differential epitope recognition might reflect host genetic differences and exposure to region-specific or unrelated pathogens [39]. Therefore, we speculated that the successful induction of T-cell responses with high magnitude and increased breadth in response to HTNV-Gn/Gc-specific T-cell epitopes might be crucial for protection against HTNV infection, consistent with the fact that HTNV-NP-specific CTLs are important for controlling viremia and disease progression in HTNV infection [18,20]. Thus, the epitopes identified herein provide the foundation for an evaluation of the role of HTNV-Gn/Gc-specific T cells in protection from HTNV infections in humans.

The main goal of this study was to determine how HTNV-Gn/Gc-specific T-cell responses eliminate virus replication in HFRS patients. However, the lack of patient blood samples prevented a detailed analysis of the 8 to 10-mer CTL epitopes and associated responses to HTNV-Gn/Gc. Therefore, we focused on the potential mechanisms of CD4^+^T-cell responses in controlling HTNV infections. The data showed that HTNV-Gn/Gc-specific CD4^+^T cells primarily produce Th1 cytokines and specific cytotoxic mediators, suggesting that the high production of antiviral cytokines is a critical characteristic of effective T-cell immunity for the suppression of virus replication and elimination of virus-infected host cells. Moreover, HTNV-Gn/Gc-specific CD4^+^T cells also displayed a high degree of polyfunctionality, simultaneously secreting IFN-γ, TNF-α or IL-2. IFN-γ has been implicated in immune regulatory and direct antiviral activities [40]. The rapid production of IFN-γ and other cytokines are important components of the T cell response against viral infections [41]. Therefore, the induction these multifunctional Th1 populations might greatly enhance the anti-HTNV immunity, associated with superior protection efficacy [42–43]. Notably, the production of Th1 cytokines is directly correlated with the IFN-γ-secreting CD8^+^T-cell response against HTNV-Gn/Gc, strongly suggesting that there are robust cellular responses induced by acute HTNV infection. The activities of Th1 cytokine-producing CD4^+^T cells may provide further benefits for expansion or function of other effector cells. However, the solid helper function of CD4^+^T cells needs to be investigated in the future study [29]. In addition to exhibiting adequate Th1 activities, a proportion of CD4^+^T cells exhibit cytotoxicity in terms of granzyme B and perforin production and the upregulation of CD107a expression upon recognition of HTNV-Gn/Gc. It has been confirmed in several studies that some CD4^+^T cells with cytotoxic potential were specifically induced at the site of infection during virus infection and implicated in the killing of virus-infected cells [32–33,44–46], and the IL-2 signaling, low antigen dose, as well as OX40 and 4-1BB stimulation may promote the development of CD4^+^T cells with cytotoxic potential [47–49]. The earlier research on SNV has demonstrated that the establishment of CD4^+^CTL clone could lyse autologous target cells pulsed with nucleoprotein peptide [14]. Importantly, the cytolytic capacity of HTNV-Gn/Gc-specific granzyme B-producing CD4^+^T cells in killing the HTNV-Gn/Gc peptides pulsed autologous B-LCLs was also observed in our experiment, with the stronger cytotoxicity percentage in much milder patients, indicating that HTNV-Gn/Gc-specific effector CD4^+^T cells against HTNV infection in HFRS patients were comprised of polyfunctional Th1 cells and cytotoxic ThGzmB^+^ cells. Although few IFN-γ^+^granzyme B^+^ double positive cells was visualized in each CD4^+^T-cell population specific to HTNV-Gn/Gn, which was similar with the findings reported in the study of *Leishmania* [50], many other studies showed the virus-specific IFN-γ ^+^CD4^+^T cells also expressed granzyme B or CD107a [51–52]. Although some researchers considered the cell-mediated cytotoxic CD4^+^T cells as a functional distinct “ThCTL” subset [32,53], it still can hardly consider that granzyme B is totally lost from the IFN-γ-secreting cells. In addition, the substantial protective effect of HTNV-Gn/Gc-specific CD4^+^T cells is also demonstrated through the inverse correlation between cytokine-producing CD4^+^T cells and the HTNV RNA load during the early phase of the disease or the apparently higher cytokine production in mild/moderate patients compared with severe/critical individuals. Furthermore, we observed CD4^+^T cell-mediated control of HTNV replication during early, but not late infection, as both IFN-γ secretion from CD4^+^T cells and peak HTNV viremia were observed during the febrile stage, followed by a gradual decrease throughout the follow-up period of the disease. Thus, the observed viremia kinetics likely reflects the putative direct antiviral effect of cytokine-producing CD4^+^T cells.

Another important observation of this study is the marked increase in CD4^+^T-cell activation and proliferation soon after HTNV infection in HFRS patients. First, the expansion of HTNV-Gn/Gc-specific CD4^+^T cells during early stage HFRS is highly associated with disease outcome. The lower percentage and higher MFI of the CFSE^dim^ cells in both CD4^+^ and CD8^+^T cells always observed in the patients with more severe HFRS, indicating there may be deficient proliferative capacity of T cells in severe/critical patients. Second, the levels of proliferating HTNV-Gn/Gc-specific CD4^+^T cells in circulation are directly correlated with the capacity of HTNV-Gn/Gc-specific CD8^+^T-cell expansion and inversely correlated with plasma HTNV viremia of the patients, suggesting that the proliferation of specific CD4^+^T cells in response to HTNV-Gn/Gc exert direct antiviral effects during HFRS. Importantly, the finding that polyclonal antibody stimulation induced great proliferative ability of T cells was stronger than the poor capacity of expansion stimulated with HTNV-Gn/Gc peptides indicated that the decrease of T-cell proliferation from the more ill individuals are refractory to HTNV-Gn/Gc, but not a systematic deficit. We may partially find the reason for this antigen-specific decline in T-cell proliferation from the different expression of inhibitory receptors on T cells during HTNV infection.

Numerous studies have examined the role of PD-1, an ITIM-containing inhibitory receptor expressed on activated T cells, and found that PD-1 on the cell cycle could lead to inhibition of T-cell expansion [54–55]. In the present study, we demonstrated that HTNV infection might differentially affect the expression of PD-1 on CD4^+^T cells. The upregulation of PD-1 expression on HTNV-Gn/Gc-specific CD4^+^T cells and the dysfunctional and senescent phenotype (CD127^lo^CD57^hi^) are more likely presented in patients with much severe HFRS. Notably, CD57 expression on T lymphocytes has been recognized as a marker of *in vitro* replicative senescence for measuring functional immune deficiency in patients with infectious diseases [56], and the upregulation of inhibitory receptors on T cells is an important mechanism of T-cell dysfunction during chronic viral infections [57]. Therefore, it is hypothesized that the higher PD-1 expressing CD4^+^T cells may account for the impairment of T-cell proliferative in more severe patients or lack of T-cell assistance during the earliest stages of infection, which determines whether CTL effectors develop into T_EM_ cells conferring immune protection. However, whether the ligands for PD-1 are highly expressed in HTNV-infected cells need to be further studied.

Active lymphocytes in different subsets could express distinct panels of lymphocyte homing receptors, reflecting differences in homing potential [58]. The early immune activation typically stimulates the production of critical T_EM_ cells and maintains the levels of T-cell differentiation and effector compartments [59]. Several studies have shown that naïve T cells and T_CM_ express CCR7, a lymph node homing receptor, for homing to secondary lymphoid organs, in which these cells proliferate and rapidly differentiate into effector cells upon encountering corresponding virus antigen [60]. Whereas, the loss of CCR7 expression on T_EM_ might generate a tissue-homing phenotype, leading to effector cell function against viruses in peripheral tissues [60–61]. Jang et al. recently showed that a predominant expansion of an activated HCV-specific CCR7^—^CTL could be observed in acute resolving HCV-infected patients, whereas HCV-specific CCR7^+^ CTLs were consistently observed in patients with persistent infections [62–63]. In particular, HFRS individuals with high plasma viral load during early stage might induce CD4^+^T_CM_ cells to differentiate into T_EM_ cells to overcome viral replication, leading to decreased viremia, reduced T_CM_ cell numbers, and much milder disease outcomes. In contrast, a partial defects or low differentiation leading to the dominant CD4^+^T_CM_ subpopulation exhibiting unsatisfactory control of the viral load upon the HTNV infection might be associated with much severe HFRS. As IFN-γ or granzyme B-producing CD4^+^T cells also showed T_EM_ phenotypes, it is tempting to hypothesize that CD4^+^T_EM_ cells lack CCR7 expression, which likely migrate to peripheral sites in the body and exert antiviral activity through the production of antiviral cytokines during the early phase of HTNV infection. Thus, the relative proportion of HTNV-Gn/Gc-specific CD4^+^T cells characterized by the ability to function as fully differentiated effectors (CD28^–^CD27^–^), acquisition of an effector tissue-homing membrane phenotype (CD45RA^–^CCR7^–^) and expression of high levels of IFN-γ or granzyme B, exhibit great effector performance and might be principally involved in determining the disease outcome of HFRS. These findings suggest that the CD4^+^T_EM_ cell subset likely serve as major effector cells against HTNV infection.

Another intriguing finding is that the expression of CD127 is downregulated on CD4^+^T cells in severe or critical patients. CD127 is known highly expressed on T cells and is considered as a marker for memory precursor CD8^+^T cells during viral infection [64]. Similar with our finding, Shin *et al*. have reported that the percentage of hepatitis C virus-specific CD127^+^T cells remained low in chimpanzees with chronically evolving hepatitis, indicating that the early expression of CD127 on T cells could predict the outcome of acute infection [65]. Furthermore, CD127 is the α chain of interleukin 7 receptor (IL-7Rα). IL-7 has been identified as a major homeostatic cytokine for mature T cell and is produced at constitutive levels by stromal cells resident in various organs, as well as by thymic and intestinal epithelial cells and fibroblastic reticular cells at T cell zone of lymph nodes [66–67]. It has been demonstrated that CD127 expression is downregulated from the cell surface upon IL-7 stimulation [68]. For example, in HIV infection, systemic levels of IL-7 are increased, whereas the decreased expression of CD127 is demonstrable on both CD4^+^ and CD8^+^T cells [69–70]. Moreover, recent study on HIV infection also showed that both IL-1β and IL-6 could decrease RNA levels and T-cell surface expression of CD127, especially on CD4^+^ T cells [71]. Based on this finding, we can speculate that the lower expression of CD127 on the CD4^+^T cells may be caused by the higher levels of proinflammatory cytokines in much more severe HFRS patients, because that the significant higher levels of IL-6 and IL-8 have been observed in more severe patients than in milder HFRS cases [72]. However, whether the decreased expression of CD127 on CD4^+^T cells in more severe patients is induced by the more IL-7 secretion during HFRS needs to be investigated in our future study.

The presence of T-cell response against HTNV infection in HFRS patients but with different clinical outcomes reveals that there may be a relationship between the role of cellular immune response in controlling HTNV infection and the mechanisms of HTNV to inhibit this response. The host-virus relationship is a dynamic process in which the virus tries to decrease its visibility, whereas the host attempts to prevent and eradicate infection [73]. On the one hand, vigorous CD4^+^ and CD8^+^T-cell responses emerge in many acute virus infections. Similar with our findings, the HCV clearance has been observed and correlated with specific CD4^+^T-cells responses, and the early priming of the CD4^+^T-cell response is required for viral clearance [74], suggesting that the stronger immune responses were necessary for control of the infection better in the milder patients. On the other hand, virus infection also affects CD4^+^T-cell responses. Recent findings showed that HCV is able to impair the cellular immune response by developing escape mutations in T-cell epitope recognition sites and inducing specific CTL anergy and deletion [75]. Besides, costimulatory molecules modulation, apoptosis induction and chemokine regulation have also been confirmed as major mechanisms of HCV to inhibit immune control [75]. Based on these, our finding that the higher expression of inhibitory molecule PD-1 on CD4^+^T cells might be one of the mechanisms mediated by the HTNV to impair the immune responses, which may lead to dysfunction of specific CD4^+^T-cell response and much more severe disease. In fact, besides the functional CD4^+^T cells we detected in peripheral blood of HFRS patients, the CD4^+^T cells are also involved in localized immune responses in tissue [76]. The pathogen activated endothelium could direct localized immune cell adherence [77]. In PUUV-infected patients, the kidney biopsies showed interstitial infiltration of lymphocytes [78], and lung biopsies showed an increase in submucosal CD4^+^ T cells [79]. Studies of fatal HPS cases also revealed mononuclear cell infiltrates in pulmonary tissue consisting largely of CD4^+^ and CD8^+^T cells [80], indicating a local immune response in terms of activated T lymphocytes. Therefore, we could speculate that the much more effective T cells may be attracted into the local tissues in more severe patients with higher viral loads, leading to a decreased number of T cells in peripheral blood to fight against the virus infection, which may partial explain the correlation between the relative lower frequency of peripheral CD4^+^T cells and the deficit function of the CD4^+^T cells in severe or critical patients.

In summary, HTNV-Gn/Gc could induce the specific CD4^+^T-cell response characterized by a broader antigenic repertoire, stronger capacity of cytotoxicity and proliferation, higher frequency of cytokine secretion and fully differentiated effector memory cell phenotype, and would elicit greater defense against HTNV infection, likely through the induction of antiviral conditions in host cells and the cell-mediated cytotoxic effects of ThGzmB^+^ cells. These findings provide insights into the mechanism of HTNV-Gn/Gc-specific CD4^+^T cells in developing an efficient anti-HTNV response in HFRS patients, thereby increasing the current understanding of the relationship between HTNV and the host immune system and evoking strong interest for further studies of T-cell immunity in HTNV infection to guide the development of future clinical therapies. However, some data we presented here are limited to the correlative analysis. In future studies, we will try to sample the sites of HTNV infection specifically and use the CD4-depletion animal models to validate the indispensability of CD4^+^T-cell immunity in protection from HTNV infection. Furthermore, whether CD4^+^T cells facilitate B cell differentiation and antibody production, particularly neutralizing antibodies against HTNV infection should also be considered next.

## Materials and Methods

### Ethics statement

Written informed consent was obtained from each HFRS patient or their guardians under a protocol approved by the Institutional Review Board of the Tangdu Hospital and the Fourth Military Medical University. The research involving human materials was also approved by the Ethical Review Board of the University, and the related information was used anonymously.

### Participants

A total of 95 HFRS patients infected with HTNV were enrolled in the study at Department of Infectious Diseases at the Tangdu Hospital of the Fourth Military Medical University (Xi’an, China). HTNV infection was confirmed via serological testing of immunoglobulin M (IgM) and IgG in serum specimens. According to the diagnostic criteria from the Prevention and Treatment Strategy of HFRS promulgated by the Ministry of Health, People’s Republic of China, the patients were classified into four clinical types [20]. Mild HFRS was defined as mild renal failure without an obvious oliguric stage and moderate for obvious symptoms of uremia, effusion (bulbar conjunctiva), hemorrhage (skin and mucous membrane), and renal failure with a typical oliguric stage. While patients with severe uremia, effusion (bulbar conjunctiva and either peritoneum or pleura), hemorrhage (skin and mucous membrane), and renal failure with oliguria (urine output, 50–500 mL/day) for ≤ 5 days or anuria (urine output, < 50 mL/day) for ≤ 2 days were defined as severe HFRS, and critical patients were considered as those with ≥ 1 of the following symptoms: refractory shock, visceral hemorrhage, heart failure, pulmonary edema, brain edema, severe secondary infection, and severe renal failure with either oliguria (urine output, 50–500 mL/day) for > 5 days, anuria (urine output, < 50 mL/day) for > 2 days, or a blood urea nitrogen level of > 42.84 mmol/L. In this case, the number of patients with a severity degree of mild, moderate, severe, and critical was 11, 24, 24 and 31, respectively. To ensure the sample size in some statistical analyses, we combined the patients according to the disease severity into mild/moderate and severe/critical groups for comparison. The patients with other kidney diseases, diabetes, cardiovascular diseases, hematological diseases, autoimmune diseases, viral hepatitis, and other liver diseases were excluded in this study. According to the clinical observation, the illness could be divided into five sequential stages: febrile, hypotensive, oliguric, diuretic, and convalescent. In general, samples were collected at 3–6 days for the febrile or hypotension stage, 7–12 days for the oliguric stage, 13–18 days for the diuretic stage and after 18 days for the convalescent stage. The phase within 8 days from the fever onset to the early oliguric stage was typically defined as acute or early phase of the disease. Moreover, eight healthy volunteers were enrolled, showing anti-HTNV negative or no HTNV risk factors, constituting the negative control group.

### Sample collection

Peripheral blood samples at different stages of HFRS were collected from each patient during hospitalization. The plasma samples of each patient were collected from heparinized whole blood before the PBMCs isolation. PBMCs were isolated using a standard Ficoll-Hypaque (Sigma-Aldrich, MO) density gradient centrifugation, and these cells were used in the following assays. The values for the clinical parameters, including platelet counts and serum creatinine levels, were regularly recorded during the hospitalization of each patient.

### Determination of plasma viral RNA

The Taqman RT-PCR assay to determine HTNV viral load was performed as previously described [34]. Briefly, viral RNA was extracted from the plasma samples of HFRS patients and the real-time RT-PCR assay was conducted. The primers and probe were designed based on the sequence alignment of the S segment of the HTNV standard strain 76–118 (NC_005218) and two hantaan virus strains, A16 (AF288646.1) and 84FLi (AY017064) obtained from Shaanxi Province. The copies of HTNV viral RNA load were log-transformed to calculate the viral load.

### Synthesis of peptides

A total of 281 15-mer peptides over-lapping 11 amino acids spanning the entire 1132 amino acid of Gn/Gc sequence of HTNV 76–118 strain (GenBank accession number: P08668.1), referred to as G1 to G281 according to the N- to C-terminal sequence, were synthesized (CL Bio-scientific, Xi’an, China) and clustered into 28 pools of 10 contiguous peptides (pools 1–28, pool 28 contained 11 peptides). Each pool covered a sequence of 51 amino acids, except pool 28, which covered sequences of 55 amino acids. All peptides were synthesized with greater than 90% purity as determined through high-performance liquid chromatography and resuspended in a sterile DMSO phosphate-buffered saline (PBS) solution at 1 mM.

### *Ex vivo* IFN-γ and granzyme B enzyme-linked immunospot (ELISPOT) assays

The identification of the HTNV-Gn/Gc specific T-cell epitopes was performed using the IFN-γ ELISPOT assay (Mabtech, Büro Deutschland, Germany) as previously described [20]. Briefly, the 28 peptide pools were firstly used to stimulate the PBMCs of HFRS patients. Subsequently, the positive response peptide pools were divided into single 15-mer peptides and used as stimuli in a second round of *ex vivo* ELISPOT assays to identify the single-positive responsed 15-mer peptides. The confirmed positive peptide was subsequently characterized through CD8 depletion of PBMCs using anti-CD8-coated Dynal beads (Invitrogen Dynal AS, Oslo, Norway). CD8^+^T-cell-depleted PBMCs were employed as effector cells for the recognition of CD4^+^T-cell responsive 15-mer epitopes. For the frequency detection of the HTNV-Gn/Gc-specific granzyme B-secreting CD4^+^T cells, the CD8^+^T cell depleted-PBMCs were further removed CD56^+^NK cells using APC labeled anti-human CD56 (Miltenyi Biotec GmbH, Germany) together with anti-APC microbeads (Miltenyi Biotec GmbH, Germany) and then used in granzyme B ELISPOT assay (Mabtech, Büro Deutschland, Germany). PBMCs or the isolated cells were placed in the ELISPOT plates at 2 × 10^5^ cells/well and stimulated with peptide pools or single peptide at a final concentration of 10 μM. Cells with phytohemagglutinin (PHA, 10 μg/mL, Sigma-Aldrich, St. Louis, MO) or no peptide stimulation served as positive and background controls, respectively. For quantification of *ex vivo* responses, the assay was duplicated. An automated ELISPOT reader (Cellular Technology Limited, USA) was used to count the spots. Adjusted spot-forming cells (SFC) after subtracting average negative values expressed as SFC/10^6^ PBMCs. The positive response was defined as at least 50 SFC/10^6^ input cells, exceeding 3 times the background response after subtracting the number of spots in the background controls from those in the stimulated samples. The SFC/10^6^ PBMCs in unstimulated control wells never exceeded 5 spots per well.

### Intracellular cytokine staining and CD107a degranulation assay

Two million freshly isolated PBMCs from each HFRS patient were stimulated for 6h with HTNV-Gn/Gc peptide pools (5 μM for each peptide) in the presence of 1 μg/ml costimulatory molecules anti-CD49d (clone 9F10) and anti-CD28 (clone CD28.2) (Biolegend), as previously described [18]. For the detection of degranulation responses to antigen stimulation, the antibody CD107a-PE (BD Pharmingen) was added to the wells during the stimulation. Cells stimulated with phorbol myristate acetate (PMA, 0.1 μg/ml, Sigma-Aldrich, St. Louis, MO)-ionomycin (0.05 μg/ml, Sigma-Aldrich, St. Louis, MO) or medium alone were used as positive or negative controls, respectively. Brefeldin A (10 μg/ml; Sigma-Aldrich, St. Louis, MO) and monensin (Golgistop, 0.7 μl/ml; BD Biosciences, San Jose, CA) were added to the culture and incubated for 4 h. After washing, the PBMCs were stained with antibodies against the surface markers, in different combinations, including CD3-PE,-APC, CD4-FITC,-PE, CD45RA-APC, CCR7-PerCP-Cy5.5, CD127-FITC, CD27-FITC, CD28-PerCP-Cy5.5, CD38-PerCP-Cy5.5, CD57-PE and CD279 (PD-1)- PerCP-Cy5.5 (BD Pharmingen) for 20 minutes at 4°C, followed by washing, fixation and permeabilization with a permeabilization buffer (BD Biosciences, San Jose, CA). The cells were subsequently stained with antibodies against intracellular markers, including IFN-γ-APC or-PE, TNF-α-PE, IL-4-PE, IL-2-FITC, granzyme B-FITC and perforin-APC (BD Pharmingen), for 15 minutes at room temperature, followed by washing, and acquisition using a FACSCalibur flow cytometer (Becton Dickinson). A total of 300,000 events per sample from the lymphocyte gate were collected for each analysis.

### Cell-mediated cytotoxicity assay

The CD4^+^T cells isolated with anti-CD4-coated Dynal beads (Invitrogen Dynal AS, Oslo, Norway) from the PBMCs of the HFRS patients were used as effector cells, and the Epstein Barr Virus (EBV) transformed autologous B lymphoblastic cell line (B-LCL) or MHC class Ⅱ partial matched B-LCL of each patient pulsed with HTNV-Gn/Gc peptides were used as target cells. A lactate dehy-drogenase (LDH) releasing Cyto Tox 96 nonradioactive cytotoxicity assay (Promega, Madison, WI, USA) was performed, as described in the manufacturer’s protocol. Briefly, CD4^+^T cells were cocultured with 5×10^3^ B-LCLs in a 96-well U-bottom cell culture plate at declining effector-to-target cell ratios of 200:1, 100:1, 50:1, 20:1, 10:1 and 5:1. After a 4-hour incubation, a 50μL supernatant of each well was transferred to a 96-well flat-bottom plate. Fifty microliters of the substrate mixture was added to each well, followed by incubation at room temperature for 30 minutes in the dark. Subsequently, 50μL of the stop solution was added to each well, and absorbance was measured at 490 nm with ELISA reader (Bio-Rad, iMark, USA). Cytotoxicity was calculated using the following formula:

%Cytotoxicity=[(Experimental−Effector Spontaneous−Target Spontaneous)/(Target Maximum−Target Spontaneous)]×100

### *Ex vivo* proliferation assay

The CFSE-labeled proliferation assay was performed as previously described [18]. Briefly, 2 × 10^7^/ml PBMCs were labeled with 10 μM 5, 6-carboxyfluorescein succinimidyl ester (CFSE, Molecular Probes, OR) at 37°C for 15 min, terminated upon the addition of fetal bovine serum and stimulated with HTNV-Gn/Gc peptide pools (5 μM). Staphylococcal enterotoxin B (SEB, 200 ng/ml, Sigma–Aldrich, MO, USA) or anti-human CD3 (Biolegend) stimulation of PBMCs served as positive controls. After 2 days, 10% exogenous IL-2 was added. After 7 days, the cells were harvested and stained with the CD3-PE or—APC (clone HIT3a), CD8-PerCP-Cy5.5 (clone RPA-T8) and CD4-APC or-PE (clone OKT4) mAbs (BD Pharmingen). Approximately 300,000 cells were acquired using a FACSCalibur (BD Immunocytometry Systems, California).

### Flow cytometric analysis

Flow cytometric analysis was performed immediately with FlowJo version 9.2 (TreeStar). FITC-, PE-, PerCP-Cy5.5- and APC-conjugated mouse IgG1, κ were used as isotype controls of the 4-color staining. Lymphocytes were defined as FSC/SSC, and CD4^+^T cells were defined as CD3^+^CD4^+^ events, displayed on an IFN-γ versus other cytokines dot plot. IFN-γ^+^ or granzyme B^+^ cells were further analyzed for expression of T-cell memory markers in a CCR7 versus CD45RA. Quadrant gates were set for T cell memory and differentiation phenotype, where naive cells were defined as CCR7^+^CD45RA^+^, central memory cells were defined as CCR7^+^CD45RA^–^, translational memory cells were defined as CCR7^–^CD45RA^+^, and effector memory cells were defined as CCR7^–^CD45RA^–^; early, intermediate and highly differentiation cells were defined as CD27^+^CD28^+^, CD27^–^CD28^+^ and CD27^–^CD28^–^, respectively. The cytokine response was considered positive when the percentage of cytokine was greater than 0.1% after background subtraction.

### Statistical analysis

Statistical analyses and graphing were performed using SPSS 16.0 (SPSS Inc., Chicago, IL, USA) and Prism software, version 5.0 (Graphpad; La Jolla, CA). The frequency of the CD4^+^ T cells and the cytokines secreted was presented as median and range values. The Wilcoxon rank sum test was used for parameter comparison between the two subject groups. For the comparison of paired design groups, the Wilcoxon's signed rank test was applied. Spearman’s rank was used as the nonparametric test for correlations between continuous variables. A two-tailed *P* value below 0.05 (*P* ≤ 0.05) was considered statistically significant.

### Accession numbers

HTNV 76–118 strain glycoprotein GenBank accession number: P08668.1; Hantaviruses used in this study: HTNV strain 76–118 (NC_005218), HTNV strain A16 (AF288646.1) and HTNV strain 84FLi (AY017064).

## Supporting Information

S1 Table T-cell responses to Hantaan virus glycoprotein peptide pools in hemorrhagic fever with renal syndrome patients. (DOCX)

S1 Fig Comparison of magnitude and breadth of HTNV-Gn/Gc-specific T-cell responses between mild and critical patients. (A-B) Comparison of (A) the total magnitudes (*y axis*) of *ex vivo* ELISPOT IFN-γ T-cell responses to the overlapping peptide pools covering the HTNV-Gn/Gc, and (B) the number of single positive responding HTNV-Gn/Gc 15-mer T-cell epitopes (*y axis*) at the acute stage between mild (n = 10) and critical patients (n = 18) (*x-axis*). Each spot represents a single patient. (C-D) Comparison of the magnitude of the epitope-specific responses (*y-axis*) of CD4^+^ (C) or CD8^+^T cells (D) at the acute stage between mild and critical patients (*x-axis*). Each spot represents a single epitope. The magnitude of the response is represented as the spot-forming cells (SFC) /10^6^ PBMCs. The Wilcoxon rank sum test was used for statistical evaluation.
(TIF)

S2 Fig The cytokine production of HTNV-Gn/Gc-specific CD4^+^T cells in mild or moderate HFRS patients. The flow cytometric plots of cytokine (IFN-γ, TNF-α, IL-2 and IL-4) and dual-cytokine (IFN-γ^+^TNF-α^+^ and IFN-γ^+^IL-2^+^)-producing HTNV-Gn/Gc-specific CD4^+^T cells in PBMCs of each mild or moderate HFRS patient during early stage infections within 8 days after disease onset. FACS contour plots were gated on CD3^+^ cells for the analysis of single intracellular cytokine and gated on CD3^+^CD4^+^ T cells for the analysis of dual intracellular-cytokine (percentages of double positive cells are shown) frequency. The numbers denote the percentage of cells within the boxed regions.
(TIF)

S3 Fig The cytokine production of HTNV-Gn/Gc-specific CD4^+^T cells in severe or critical HFRS patients. The flow cytometric plots of cytokine (IFN-γ, TNF-α, IL-2 and IL-4) and dual-cytokine (IFN-γ^+^TNF-α^+^ and IFN-γ^+^IL-2^+^)-producing HTNV-Gn/Gc-specific CD4^+^T cells in PBMCs of each severe or critical HFRS patient during early stage infections within 8 days after disease onset. FACS contour plots were gated on CD3^+^ cells for the analysis of single intracellular cytokine and gated on CD3^+^CD4^+^ T cells for the analysis of dual intracellular-cytokine (percentages of double positive cells are shown) frequency. The numbers denote the percentage of cells within the boxed regions.
(TIF)

S4 Fig The cytotoxic mediators production of HTNV-Gn/Gc-specific CD4^+^T cells in mild or moderate HFRS patients. The flow cytometric plots of cytotoxic mediator (granzyme B and perforin)-producing and CD107a-expressing CD4^+^T cells in PBMCs and granzyme B^+^CD107a^+^ HTNV-Gn/Gc-specific CD4^+^T cells in each mild or moderate patient during early stage infections. FACS contour plots were gated on CD3^+^ cells for the analysis of single mediator and gated on CD3^+^CD4^+^ T cells for the analysis of dual mediators (percentages of double positive cells are shown) frequency. The numbers denote the percentage of cells within the boxed regions. Gran B, granzyme B.
(TIF)

S5 Fig The cytotoxic mediators production of HTNV-Gn/Gc-specific CD4^+^T cells in severe or critical HFRS patients. The flow cytometric plots of cytotoxic mediator (granzyme B and perforin)-producing and CD107a-expressing CD4^+^T cells in PBMCs and granzyme B^+^CD107a^+^ HTNV-Gn/Gc-specific CD4^+^T cells during early stage infections in each severe or critical HFRS patient during early stage infections. FACS contour plots were gated on CD3^+^ cells for the analysis of single mediator and gated on CD3^+^CD4^+^ T cells for the analysis of dual mediators (percentages of double positive cells are shown) frequency. The numbers denote the percentage of cells within the boxed regions. Gran B, granzyme B.
(TIF)

S6 Fig The granzyme B production of HTNV-Gn/Gc-specific CD4^+^T cells in ELISPOT assay. The CD8^+^T cells and CD56^+^NK cells depleted-PBMCs were used as effector cells in each well stimulated by the HTNV-Gn/Gc peptide pools. phytohemagglutinin (PHA) or no peptide stimulation served as positive and background controls, respectively. The *left lanes* (A) present the mild/moderate HFRS patients (n = 11) and the *right lanes* (B) present the severe/critical individuals (n = 11).
(TIF)

S7 Fig The lysis kinetics of peptides-pulsed target cells by HTNV-Gn/Gc-specific CD4^+^T cells in each patient. The *left lanes* present the mild/moderate HFRS patients (n = 7) and the *right lanes* present the severe/critical individuals (n = 9). The CD4^+^T cells isolated from the PBMCs of the HFRS patients were used as effector cells, and the Epstein Barr Virus (EBV) transformed autologous B lymphoblastic cell line (B-LCL) or MHC class Ⅱ partial matched B-LCL of each patient pulsed with HTNV-Gn/Gc peptides were used as target cells. The effector-to-target ratios included 200:1, 100:1, 50:1, 20:1, 10:1 and 5:1. The dark spots and the circles represent the lysis percentage of CD4^+^T cells to kill the HTNV-Gn/Gc peptides-pulsed target cells in mild/moderate and severe/critical patients, respectively. The triangles represent the lysis percentage of CD4^+^T cells from the patient to kill no peptide-pulsed target cells.
(TIF)

S8 Fig The proliferation capacity of HTNV-Gn/Gc-specific T cells in each HFRS patient. The flow cytometric plots of the expansion percentage of HTNV-Gn/Gc-specific CD4^+^ or CD8^+^T cells during acute HFRS in mild/moderate (*left lanes*) or severe/critical patients (*right lanes*) (n = 10 respectively). The expansion extent of the HTNV-Gn/Gc-specific CD4^+^ or CD8^+^T cells is shown in the *upper left* quadrants of each figure, reflecting the decrease of CFSE in the dividing CD4^+^ or CD8^+^T cells. The numbers denote the percentage of cells within the boxed regions.
(TIF)

S9 Fig The expansion capacity of T cells stimulated by HTNV-Gn/Gc or SBE in HFRS patients. The flow cytometric plot of the expansion percentage of CD4^+^ or CD8^+^T cells stimulated by the HTNV-Gn/Gc or polyclonal activator SEB control during acute HFRS in each patient. The expansion extent of the HTNV-Gn/Gc-specific CD4^+^ or CD8^+^T cells is shown in the *upper left* quadrants of each figure. The numbers denote the percentage of cells within the boxed regions. The overlay of the three conditions in histograms showed that the CFSE curve of both CD4^+^ and CD8^+^T cells from the SEB stimulation is shifted more to the right than that stimulated by the HTNV-Gn/Gc.
(TIF)

S10 Fig Comparison of the expansion capacity of T cells between the HTNV-Gn/Gc stimulation and SEB control. The percentages of CFSE^dim^ CD4^+^ (A) or CD8^+^ (B) T cells at acute stage of HFRS were compared between HTNV-Gn/Gc stimulation group and polyclonal activator SEB control group. Wilcoxon's signed rank test was used for statistical evaluation.
(TIF)

S11 Fig Comparison of the CD8^+^T-cell expansion capacity between the two groups of HFRS patients. Comparison of the percentage (A) and MFI (B) of CFSE^dim^ CD8^+^T cells stimulated by HTNV-Gn/Gc at the acute phase of HFRS between mild/moderate and severe/critical patients. The Wilcoxon rank sum test was used for statistical evaluation.
(TIF)
