# Supplementary material for: Hantaan Virus Infection Induces Both Th1 and ThGranzyme B+ Cell Immune Responses That Associated with Viral Control and Clinical Outcome in Humans
Source: PLoS Pathog. 2015 Apr 2;11(4):e1004788. doi: 10.1371/journal.ppat.1004788 (PMC4383613; doi:10.1371/journal.ppat.1004788)
Supplement: S1 Table — (DOCX) [file ppat.1004788.s001.docx]

**Table S1** T cell responses to Hantaan virus glycoprotein peptide pools in hemorrhagic fever with renal syndrome patients

| Glycoprotein | Peptide pools | 15-mer peptides containing | Amino acid  position | Number of positively responding patients | % (/70 patients) | Spots/10^6^PBMC |
| --- | --- | --- | --- | --- | --- | --- |
| Gn | P1 | G1- G10 | aa1-aa51 | 19 | 27.14 | 50-1000 |
|  | P2 | G11- G20 | aa41-aa91 | 2 | 2.86 | 170-760 |
|  | P3 | G21- G30 | aa81-aa131 | 4 | 5.71 | 50-90 |
|  | P4 | G31- G40 | aa121-aa171 | 12 | 17.14 | 60-490 |
|  | P5^a^ | G41- G50^a^ | aa161-aa211^a^ | 21^a^ | 30.00^a^ | 50-590^a^ |
|  | P6 | G51- G60 | aa201-aa251 | 1 | 1.43 | 210 |
|  | P7 | G61- G70 | aa241-aa291 | 13 | 18.57 | 50-670 |
|  | P8 | G71- G80 | aa281-aa331 | 16 | 22.86 | 50-580 |
|  | P9 | G81- G90 | aa321-aa371 | 1 | 1.43 | 70 |
|  | P10 | G91- G100 | aa361-aa411 | 4 | 5.71 | 50-300 |
|  | P11 | G101- G110 | aa401-aa451 | 5 | 7.14 | 50-530 |
|  | P12^a^ | G111- G120^a^ | aa441-aa491^a^ | 25^a^ | 35.71^a^ | 50-820^a^ |
|  | P13 | G121- G130 | aa481-aa531 | 8 | 11.43 | 60-280 |
|  | P14 | G131- G140 | aa521-aa571 | 11 | 15.71 | 50-160 |
|  | P15 | G141- G150 | aa561-aa611 | 5 | 7.14 | 60-460 |
|  | P16 | G151- G160 | aa601-aa651 | 7 | 10.00 | 50-240 |
| Gc | P17^b^ | G161- G170^b^ | aa641-aa691^b^ | 11^b^ | 15.71^b^ | 50-990^b^ |
|  | P18^b^ | G171- G180^b^ | aa681-aa731^b^ | 11^b^ | 15.71^b^ | 50-670^b^ |
|  | P19^b^ | G181- G190^b^ | aa721-aa771^b^ | 20^b^ | 28.57^b^ | 50-1300^b^ |
|  | P20^*^ | G191- G200^b^ | aa761-aa811^b^ | 18^b^ | 25.71^b^ | 50-420^b^ |
|  | P21^a,b^ | G201- G210^a,b^ | aa801-aa851^a,b^ | 29^a,b^ | 41.43^a,b^ | 50-590^a,b^ |
|  | P22^b^ | G211- G220^b^ | aa841-aa891^b^ | 8^b^ | 11.43^b^ | 60-320^b^ |
|  | P23^b^ | G221- G230^b^ | aa881-aa931^b^ | 8^b^ | 11.43^b^ | 50-480^b^ |
|  | P24 | G231- G240 | aa921-aa971 | 3 | 4.29 | 180-240 |
|  | P25^a^ | G241- G250^a^ | aa961-aa1101^a^ | 24^a^ | 34.29^a^ | 50-780^a^ |
|  | P26 | G251- G260 | aa1001-aa1051 | 1 | 1.43 | 70 |
|  | P27 | G261- G270 | aa1041-aa1091 | 5 | 7.14 | 50-400 |
|  | P28 | G271- G281 | aa1081-aa1135 | 14 | 20.00 | 50-580 |

^a^ Peptide pools frequently recognized in more than 30% of the subjects.

^b^ The most frequently recognized and strongest responding HTNV glycoprotein-reactive T cell peptide pools.
