# Supplementary figures and images for: Hantaan Virus Infection Induces Both Th1 and ThGranzyme B+ Cell Immune Responses That Associated with Viral Control and Clinical Outcome in Humans
Source: PLoS Pathog. 2015 Apr 2;11(4):e1004788. doi: 10.1371/journal.ppat.1004788 (PMC4383613; doi:10.1371/journal.ppat.1004788)

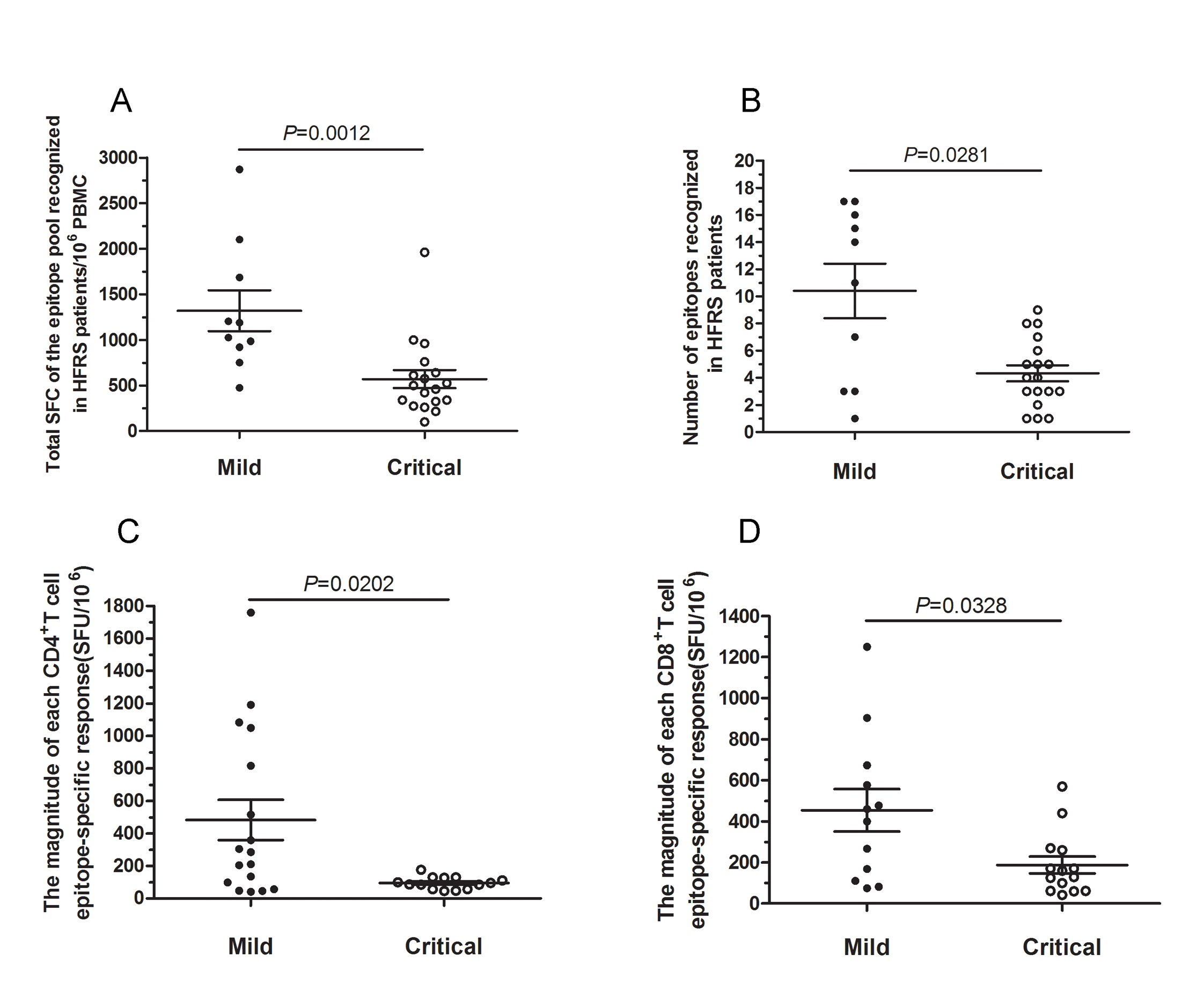

Supplement: S1 Fig — (A-B) Comparison of (A) the total magnitudes (y axis) of ex vivo ELISPOT IFN-γ T-cell responses to the overlapping peptide pools covering the HTNV-Gn/Gc, and (B) the number of single positive responding HTNV-Gn/Gc 15-mer T-cell epitopes (y axis) at the acute stage between mild (n = 10) and critical patients (n = 18) (x-axis). Each spot represents a single patient. (C-D) Comparison of the magnitude of the epitope-specific responses (y-axis) of CD4+ (C) or CD8+T cells (D) at the acute stage between mild and critical patients (x-axis). Each spot represents a single epitope. The magnitude of the response is represented as the spot-forming cells (SFC) /106 PBMCs. The Wilcoxon rank sum test was used for statistical evaluation. (TIF) [file ppat.1004788.s002.tif]

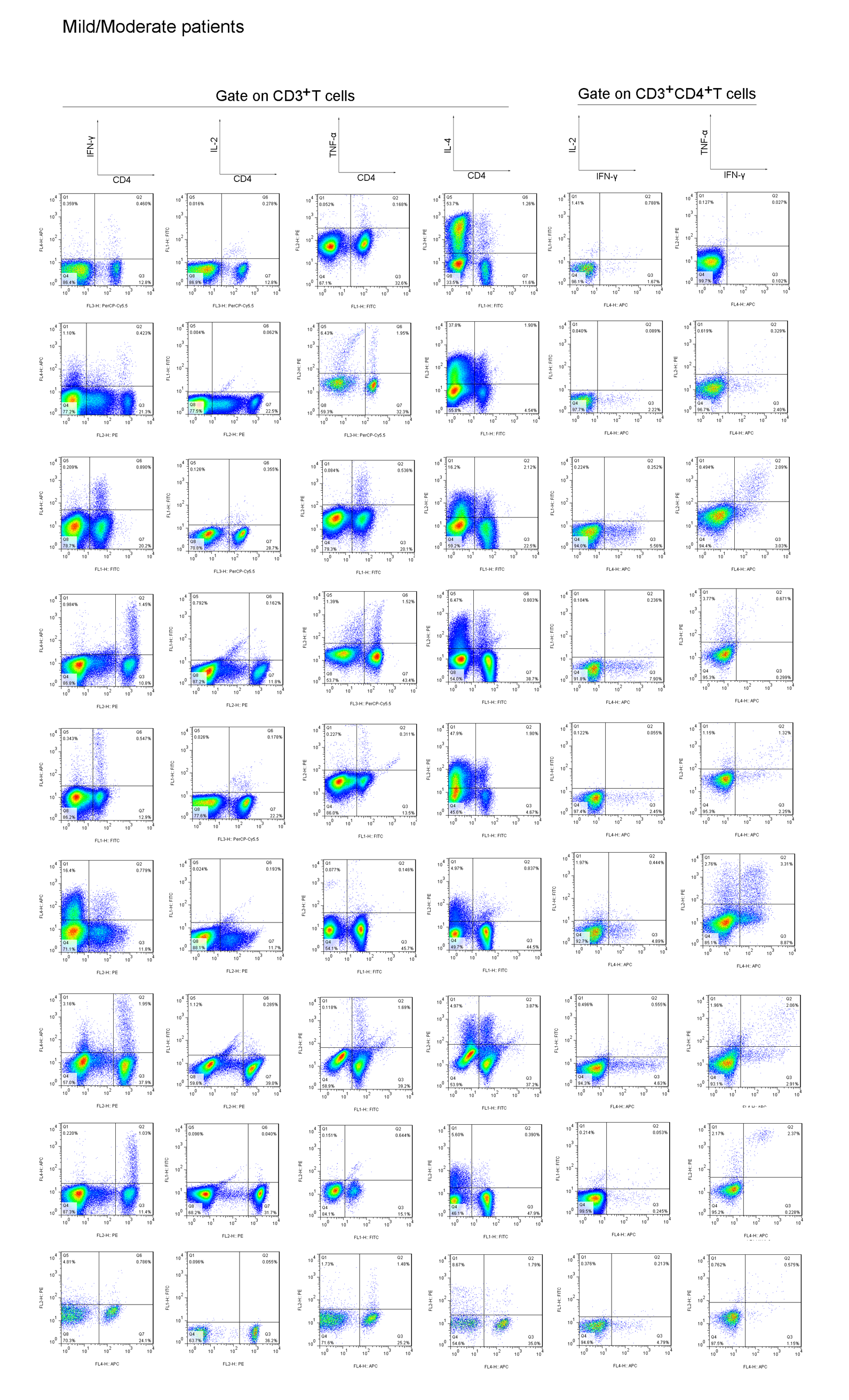

Supplement: S2 Fig — The flow cytometric plots of cytokine (IFN-γ, TNF-α, IL-2 and IL-4) and dual-cytokine (IFN-γ+TNF-α+ and IFN-γ+IL-2+)-producing HTNV-Gn/Gc-specific CD4+T cells in PBMCs of each mild or moderate HFRS patient during early stage infections within 8 days after disease onset. FACS contour plots were gated on CD3+ cells for the analysis of single intracellular cytokine and gated on CD3+CD4+ T cells for the analysis of dual intracellular-cytokine (percentages of double positive cells are shown) frequency. The numbers denote the percentage of cells within the boxed regions. (TIF) [file ppat.1004788.s003.tif]

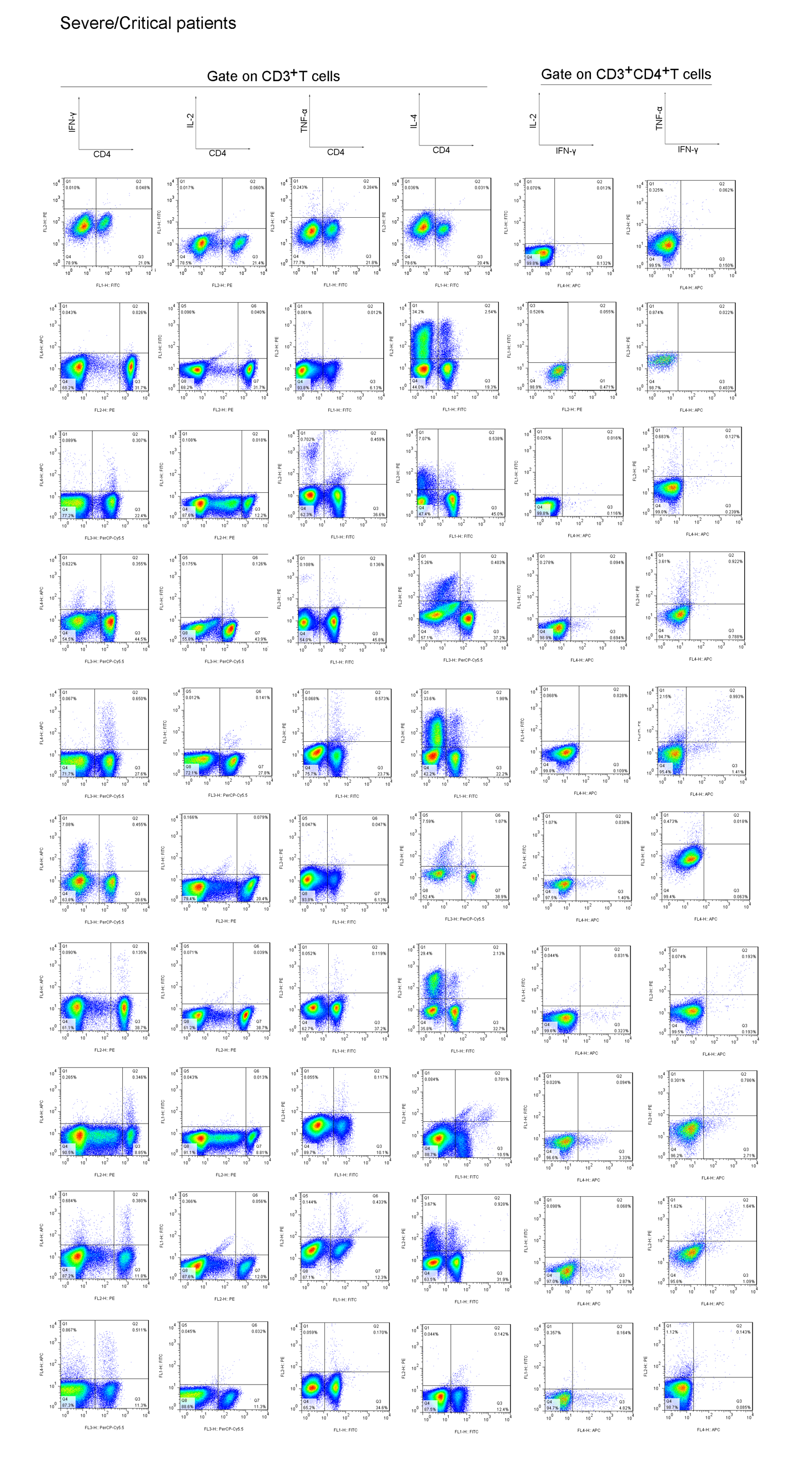

Supplement: S3 Fig — The flow cytometric plots of cytokine (IFN-γ, TNF-α, IL-2 and IL-4) and dual-cytokine (IFN-γ+TNF-α+ and IFN-γ+IL-2+)-producing HTNV-Gn/Gc-specific CD4+T cells in PBMCs of each severe or critical HFRS patient during early stage infections within 8 days after disease onset. FACS contour plots were gated on CD3+ cells for the analysis of single intracellular cytokine and gated on CD3+CD4+ T cells for the analysis of dual intracellular-cytokine (percentages of double positive cells are shown) frequency. The numbers denote the percentage of cells within the boxed regions. (TIF) [file ppat.1004788.s004.tif]

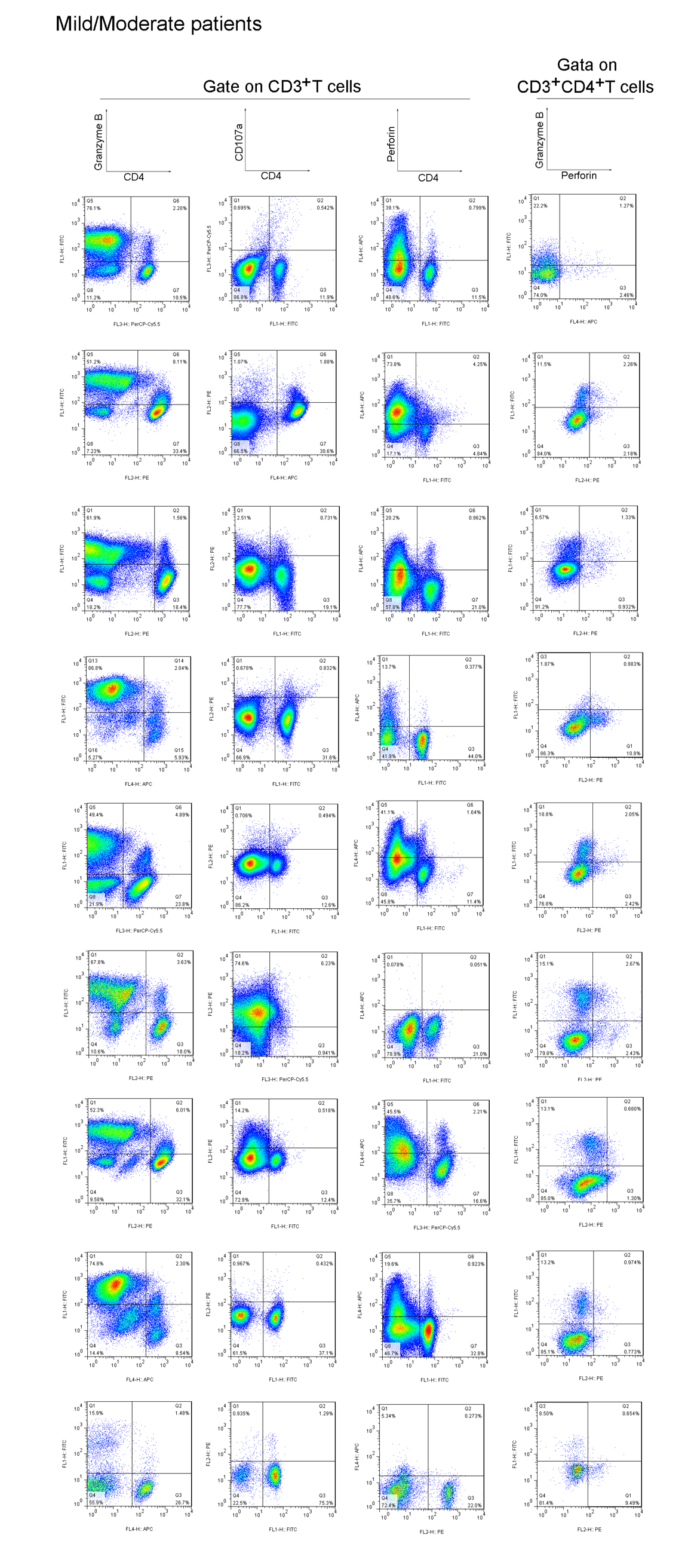

Supplement: S4 Fig — The flow cytometric plots of cytotoxic mediator (granzyme B and perforin)-producing and CD107a-expressing CD4+T cells in PBMCs and granzyme B+CD107a+ HTNV-Gn/Gc-specific CD4+T cells in each mild or moderate patient during early stage infections. FACS contour plots were gated on CD3+ cells for the analysis of single mediator and gated on CD3+CD4+ T cells for the analysis of dual mediators (percentages of double positive cells are shown) frequency. The numbers denote the percentage of cells within the boxed regions. Gran B, granzyme B. (TIF) [file ppat.1004788.s005.tif]

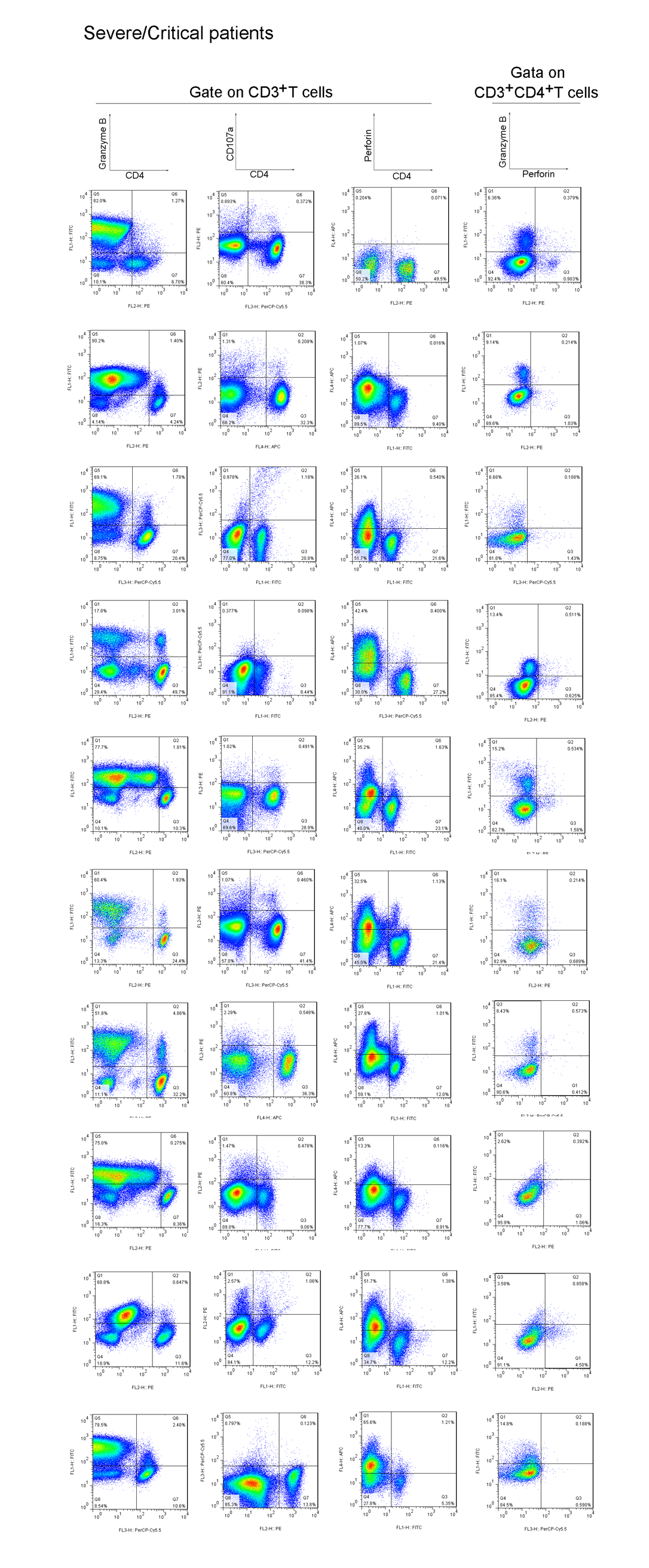

Supplement: S5 Fig — The flow cytometric plots of cytotoxic mediator (granzyme B and perforin)-producing and CD107a-expressing CD4+T cells in PBMCs and granzyme B+CD107a+ HTNV-Gn/Gc-specific CD4+T cells during early stage infections in each severe or critical HFRS patient during early stage infections. FACS contour plots were gated on CD3+ cells for the analysis of single mediator and gated on CD3+CD4+ T cells for the analysis of dual mediators (percentages of double positive cells are shown) frequency. The numbers denote the percentage of cells within the boxed regions. Gran B, granzyme B. (TIF) [file ppat.1004788.s006.tif]

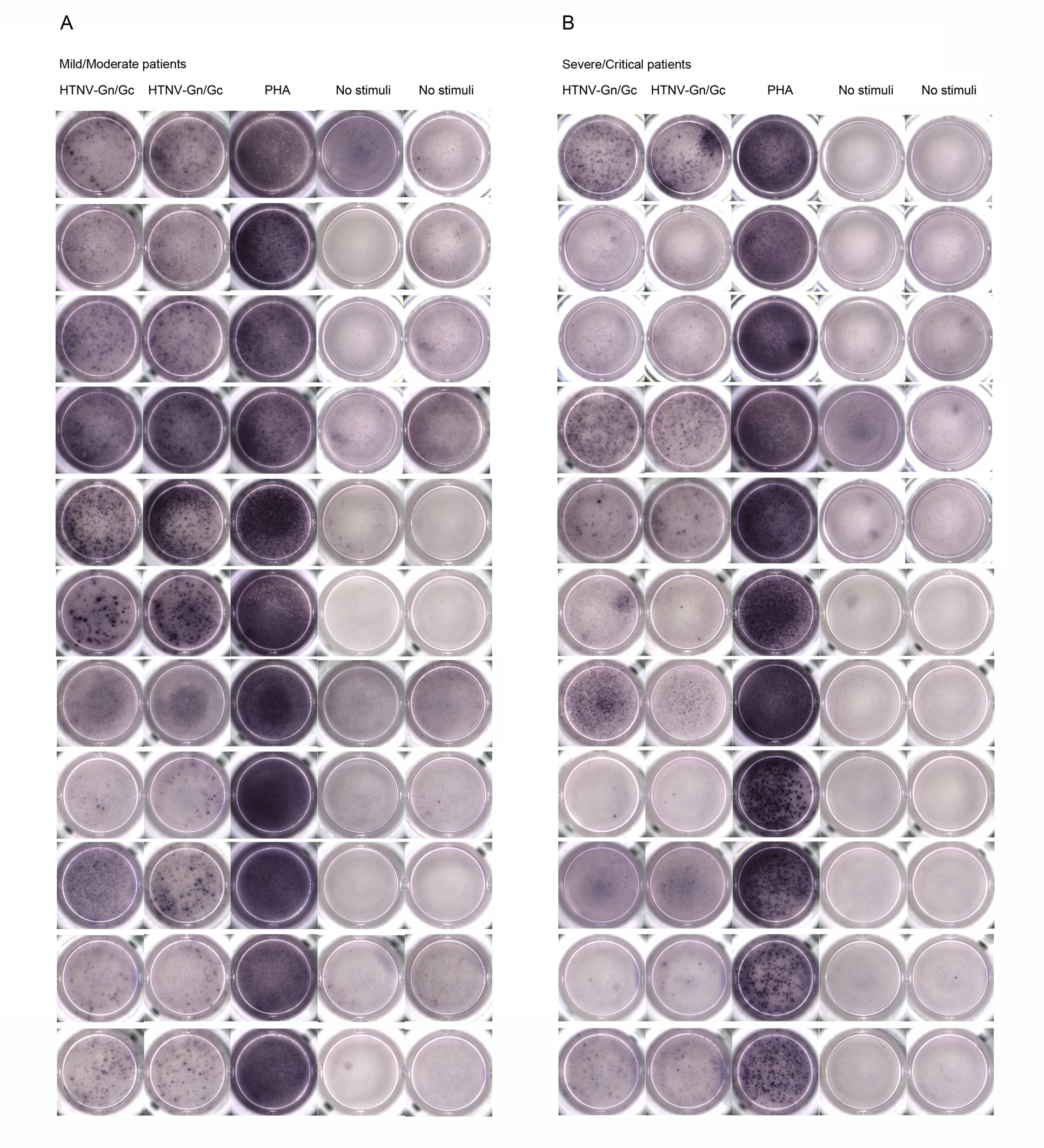

Supplement: S6 Fig — The CD8+T cells and CD56+NK cells depleted-PBMCs were used as effector cells in each well stimulated by the HTNV-Gn/Gc peptide pools. phytohemagglutinin (PHA) or no peptide stimulation served as positive and background controls, respectively. The left lanes (A) present the mild/moderate HFRS patients (n = 11) and the right lanes (B) present the severe/critical individuals (n = 11). (TIF) [file ppat.1004788.s007.tif]

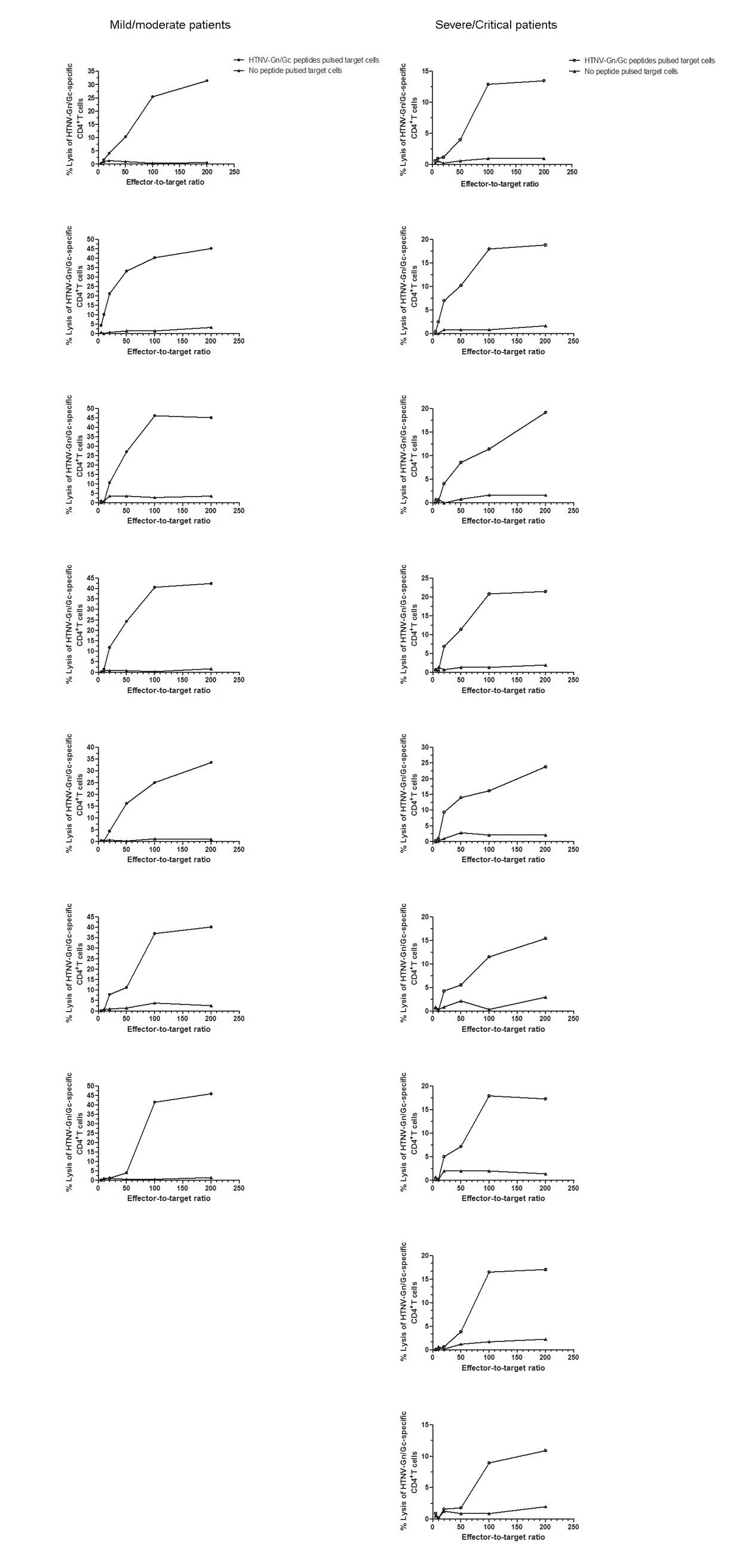

Supplement: S7 Fig — The left lanes present the mild/moderate HFRS patients (n = 7) and the right lanes present the severe/critical individuals (n = 9). The CD4+T cells isolated from the PBMCs of the HFRS patients were used as effector cells, and the Epstein Barr Virus (EBV) transformed autologous B lymphoblastic cell line (B-LCL) or MHC class Ⅱ partial matched B-LCL of each patient pulsed with HTNV-Gn/Gc peptides were used as target cells. The effector-to-target ratios included 200:1, 100:1, 50:1, 20:1, 10:1 and 5:1. The dark spots and the circles represent the lysis percentage of CD4+T cells to kill the HTNV-Gn/Gc peptides-pulsed target cells in mild/moderate and severe/critical patients, respectively. The triangles represent the lysis percentage of CD4+T cells from the patient to kill no peptide-pulsed target cells. (TIF) [file ppat.1004788.s008.tif]

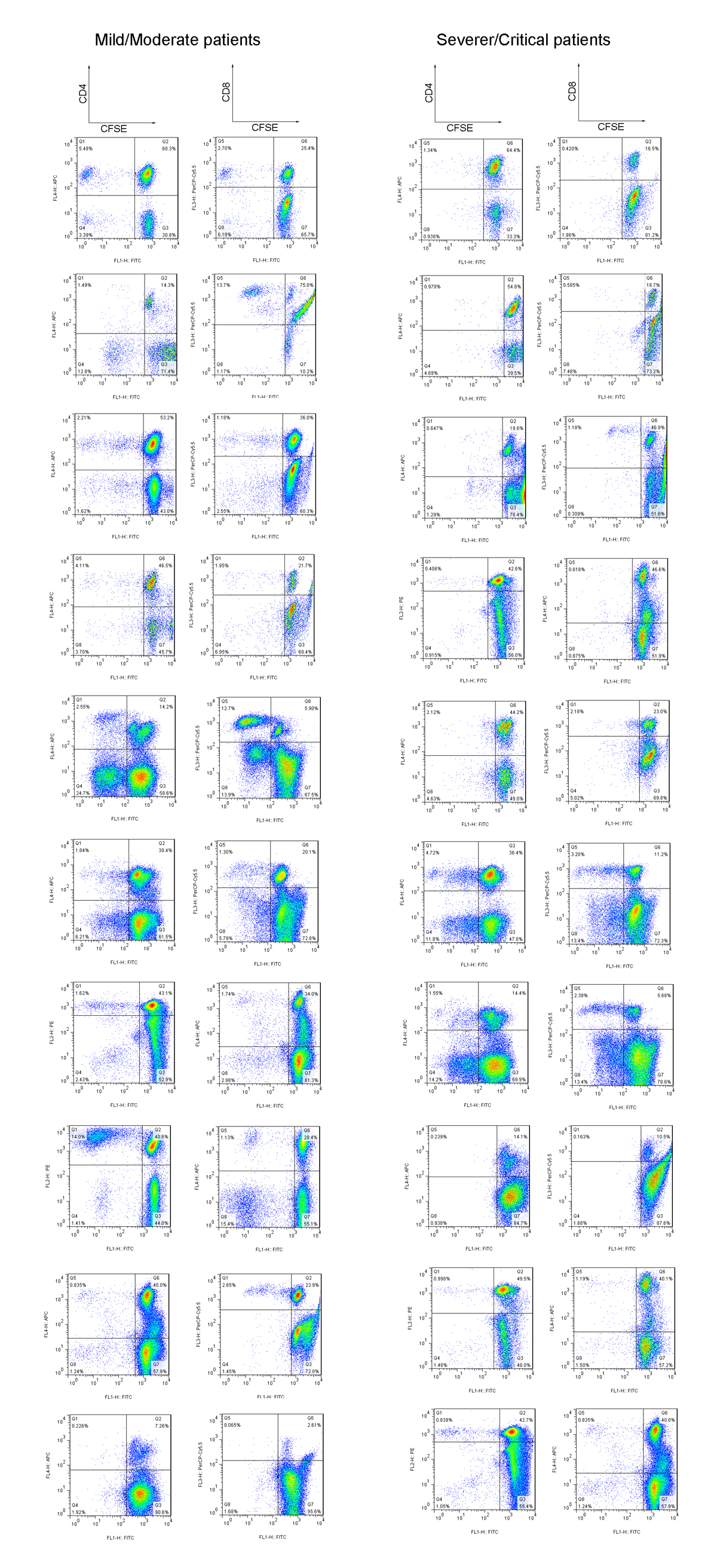

Supplement: S8 Fig — The flow cytometric plots of the expansion percentage of HTNV-Gn/Gc-specific CD4+ or CD8+T cells during acute HFRS in mild/moderate (left lanes) or severe/critical patients (right lanes) (n = 10 respectively). The expansion extent of the HTNV-Gn/Gc-specific CD4+ or CD8+T cells is shown in the upper left quadrants of each figure, reflecting the decrease of CFSE in the dividing CD4+ or CD8+T cells. The numbers denote the percentage of cells within the boxed regions. (TIF) [file ppat.1004788.s009.tif]

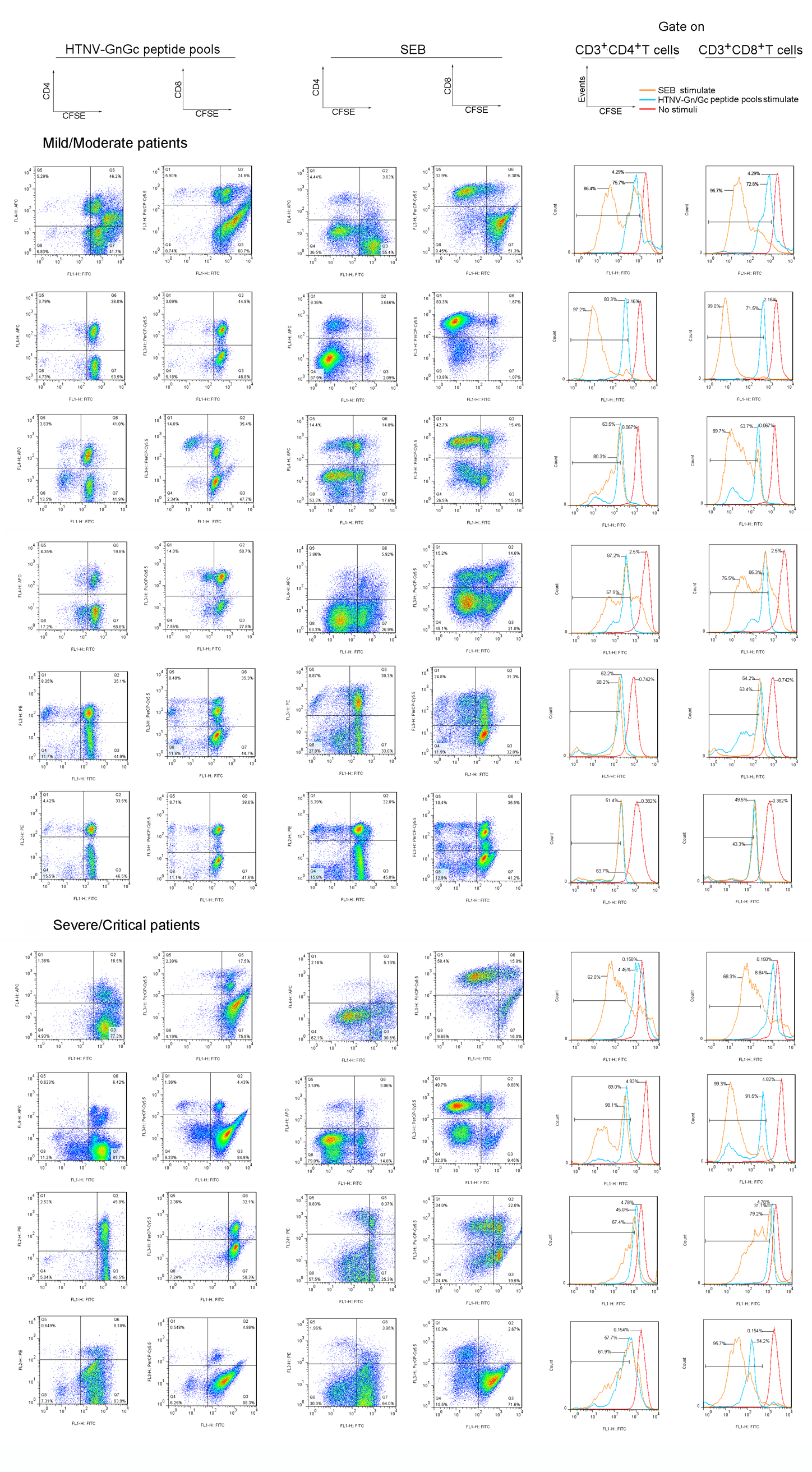

Supplement: S9 Fig — The flow cytometric plot of the expansion percentage of CD4+ or CD8+T cells stimulated by the HTNV-Gn/Gc or polyclonal activator SEB control during acute HFRS in each patient. The expansion extent of the HTNV-Gn/Gc-specific CD4+ or CD8+T cells is shown in the upper left quadrants of each figure. The numbers denote the percentage of cells within the boxed regions. The overlay of the three conditions in histograms showed that the CFSE curve of both CD4+ and CD8+T cells from the SEB stimulation is shifted more to the right than that stimulated by the HTNV-Gn/Gc. (TIF) [file ppat.1004788.s010.tif]

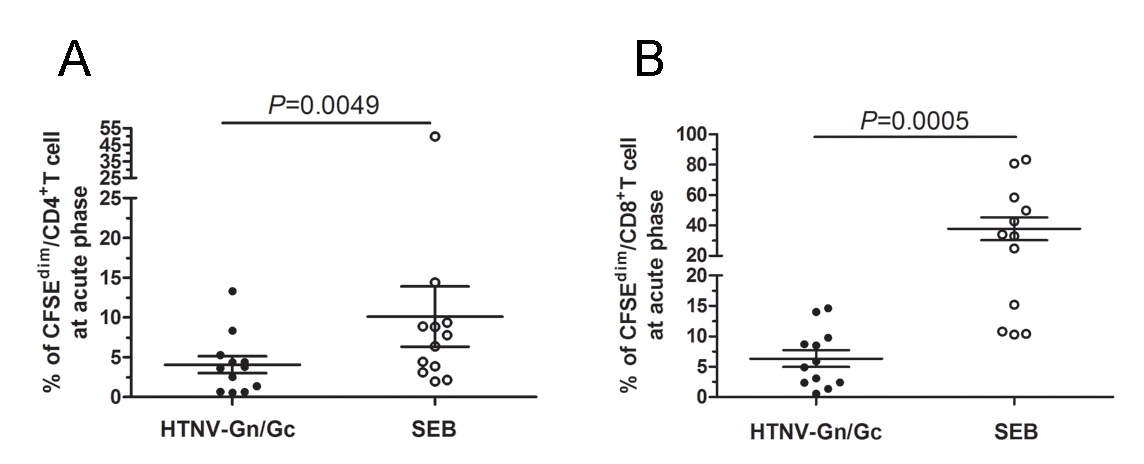

Supplement: S10 Fig — The percentages of CFSEdim CD4+ (A) or CD8+ (B) T cells at acute stage of HFRS were compared between HTNV-Gn/Gc stimulation group and polyclonal activator SEB control group. Wilcoxon's signed rank test was used for statistical evaluation. (TIF) [file ppat.1004788.s011.tif]

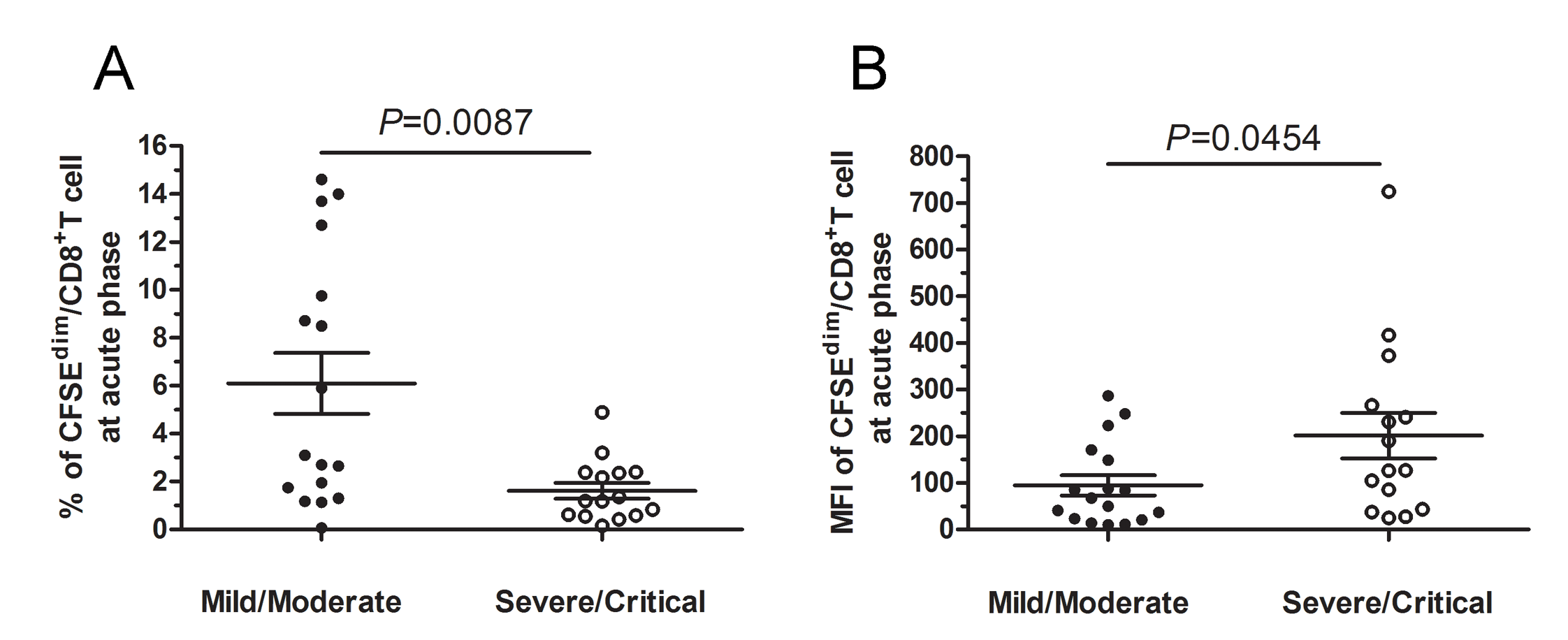

Supplement: S11 Fig — Comparison of the percentage (A) and MFI (B) of CFSEdim CD8+T cells stimulated by HTNV-Gn/Gc at the acute phase of HFRS between mild/moderate and severe/critical patients. The Wilcoxon rank sum test was used for statistical evaluation. (TIF) [file ppat.1004788.s012.tif]
